# Screening and Whole-Genome Analysis of Probiotic Lactic Acid Bacteria with Potential Antioxidants from Yak Milk and Dairy Products in the Qinghai–Tibet Plateau

**DOI:** 10.3390/antiox14020173

**Published:** 2025-01-31

**Authors:** Diyan Wu, Haichuan Li, Xuan Wang, Runtong Chen, Di Gong, Danfeng Long, Xiaodan Huang, Zhenchuang Tang, Ying Zhang

**Affiliations:** 1 School of Public Health, Lanzhou University, Lanzhou 730000, China; 220220912960@lzu.edu.cn (D.W.); lhcj0528@163.com (H.L.); wangxuan20@lzu.edu.cn (X.W.); 13822987869@163.com (R.C.); gongd@lzu.edu.cn (D.G.); longdf@lzu.edu.cn (D.L.); huangxiaodan@lzu.edu.cn (X.H.); 2 Institute of Food and Nutrition Development, Ministry of Agriculture and Rural Affairs, Beijing 100000, China

**Keywords:** lactic acid bacteria, antioxidant, probiotic properties, whole-genome sequencing, Qinghai–Tibet Plateau

## Abstract

This study aimed to isolate lactic acid bacteria (LAB) with strong antioxidant activity and potential probiotic properties from yak milk and dairy products in the Qinghai–Tibet Plateau. Initial screening of the isolates was performed using the 2,2-diphenyl-1-picrylhydrazyl (DPPH) scavenging assay and a hydrogen peroxide tolerance test. Subsequently, the antioxidant capacity of the isolates was assessed through five distinct assays: 2,2′-azino-bis (3-ethylbenzthiazoline)-6-sulfonic acid (ABTS) radical scavenging ability, superoxide anion radical scavenging ability, hydroxyl radical scavenging ability, a DPPH scavenging assay, and a reducing activity assay. The strains with the stronger antioxidant potential were then further evaluated for their probiotic properties. Whole-genome sequencing was conducted on *Lactobacillus plantarum* QL01. Among 1205 isolates, 9 strains exhibited potential antioxidant capabilities. Following probiotic property evaluation, QL01 was identified as a safe candidate due to its strong growth, strong adhesion ability, and resilience to acidic, bile, and simulated gastrointestinal conditions. Genome analysis revealed that most of QL01’s genes were involved in carbohydrate metabolism. Further examination of antibiotic resistance and virulence factors confirmed its safety, meanwhile genes linked to adhesion and stress responses underscored its probiotic potential. In conclusion, QL01, a strong antioxidant strain, was successfully isolated, and its probiotic potential was confirmed through comprehensive in vitro and genomic analyses.

## 1. Introduction

Oxidative stress refers to the excessive production of free radicals and reactive oxygen species (ROS) within the body, typically triggered by exposure to various harmful stimuli. When oxidative processes surpass the body’s antioxidant defenses, an imbalance arises between pro-oxidant and antioxidant systems, leading to cellular damage, tissue injury, and the initiation of inflammatory responses [1]. The accumulation of ROS has also been implicated in aging and the development of chronic diseases such as atherosclerosis and liver cirrhosis [2]. Antioxidants play a crucial role in mitigating oxidative stress, and as a result, synthetic antioxidants and antioxidant-rich foods are often consumed to counteract these effects. However, chemically synthesized antioxidants may carry health risks [3]. Consequently, identifying and developing cost-effective, efficient, safe, and non-toxic natural antioxidants from biological sources remains a critical priority.

Lactic acid bacteria (LAB) are a group of microorganisms capable of efficiently fermenting carbohydrates to produce significant amounts of lactic acid. Due to their notable antioxidant properties, LAB have emerged as a promising natural source of antioxidants that are accessible and safe [4]. However, the antioxidant capacity of LAB varies considerably between strains from different sources [5], highlighting the need for the isolation and screening of LAB strains with high antioxidant potential, which has become a focus of recent research.

Yak milk and its products are an important part of the traditional dietary pattern for Tibetan pastoralists in the Qinghai–Tibet Plateau. These products are rich in LAB, making them a valuable source of probiotics [6]. Due to the unique environmental conditions of the Qinghai–Tibet Plateau characterized by low temperatures, low pressure, high altitude, hypoxia, and substantial temperature fluctuations between day and night, LAB from these foods have adapted to thrive under these harsh conditions. This adaptation likely enhances their biological performance, including their antioxidant properties [7].

The objectives of this study were to screen for LAB with high antioxidant activity from yak milk and dairy products in the Qinghai–Tibet Plateau. Additionally, the study aimed to assess the safety and physiological characteristics of the potential probiotic strains through whole-genome sequencing and bioinformatic analysis. The findings are intended to provide a critical scientific foundation for the establishment and utilization of a specific LAB germplasm library for yak milk and dairy products produced under the extreme environmental conditions of the Qinghai–Tibet Plateau. Furthermore, the study provides a theoretical basis for the functional exploration of probiotics derived from the Qinghai–Tibet Plateau and will promote the application of probiotics in the field of antioxidant health food and pharmaceuticals.

## 2. Materials and Methods

### 2.1. Strain Isolation, Purification, and Identification

A total of 115 food samples, including yak milk, yogurt, milk residue, and others, were collected from diverse ecological and geographical regions in the Qinghai–Tibet Plateau. LAB were isolated using the method by Rai et al. (2022) [8]. Samples (1 mL) were added to 9 mL of sterile normal saline (0.9% NaCl) and mixed thoroughly. The samples were then gradient-diluted 10^−2^, 10^−3^, 10^−4^, 10^−5^, 10^−6^, and 10^−7^ times using sterile normal saline. Subsequently, 200 μL of the diluted sample from the 10^−4^ to 10^−7^ dilutions was spread on de Man, Rogosa, and Sharpe (MRS) medium (OXOID Biotechnology Ltd., Basingstoke, UK) and incubated at 37 °C for 48–72 h. The strains were then isolated, purified, and identified using a high throughput screening system, including the QPix selection system (Qpix 420F, Molecular Devices, San Jose, CA, USA) combined with fully automated rapid microbial mass spectrometry (MBT Sirius, Bruker, Saarbrucken, Germany).

### 2.2. Large-Scale Screening of Strains with Antioxidant Properties

#### 2.2.1. 2,2-Diphenyl-1-Picrylhydrazyl (DPPH) Radical Scavenging Ability

The method described by Zhang et al. (2022) [9] was followed with minor adjustments. The fermentation broth of strains was adjusted to a concentration of 1 × 10^8^ CFU/mL in MRS broth. Next, 1 mL of the sample was mixed with 1 mL of 0.2 mmol/L DPPH ethanol solution, followed by agitation and incubation in the dark at room temperature for 30 min. After incubation, the mixture was centrifuged at 4000 rpm for 10 min at 4 °C. The absorbance of the supernatant was measured at 517 nm. In the control group, the samples were replaced with an equal volume of sterile normal saline, while in the blank group, DPPH was replaced with an equal volume of absolute alcohol. The antioxidant activity was calculated as follows:

(1) $$\mathrm{DPPH}\;\mathrm{radical}\;\mathrm{scavenging}\;\mathrm{rate}\;\left(\%\right)=\left[1-\frac{A_{1}-A_{2}}{A_{0}}\right]\times 100\%$$

A_0_: The absorbance of saline and DPPH solution; A_1_: the absorbance of the sample and the DPPH solution; A_2_: the absorbance of the sample and the absolute alcohol solution.

#### 2.2.2. Assay of Hydrogen Peroxide Resistance

The selected strains were inoculated at 3% (*v*/*v*) into MRS broth containing initial hydrogen peroxide concentrations of 0, 1, 2, and 3 mmol/L, and incubated at 37 °C for 24 h. The OD_600_ nm values were measured after incubation, according to the method by Cele et al. (2022) [10].

### 2.3. Fine-Grained Assessment of the Antioxidant Properties

#### 2.3.1. Preparation of Cell-Free Fermentation Supernatants (CFSs), Intact Cells (ICs), and Cell-Free Extracts (CFEs) of Strains

The strains were cultured in MRS broth at 37 °C for 24 h. After incubation, the cultures were centrifuged at 10,000 rpm for 15 min at 4 °C to collect the supernatant, which was then filtered through a 0.22 μm membrane to obtain the cell-free supernatants. Meanwhile, the cell pellets were washed three times with sterile phosphate-buffered saline (PBS, pH 7.4) and resuspended in PBS to a final concentration of 10^9^ CFU/mL. This bacterial suspension was then subjected to cell disruption in an ultrasonic ice bath (6 mm probe, 5 s on/off cycles, 300 W, for 30 min). The resulting supernatant, obtained after centrifugation at 8000 rpm for 10 min at 4 °C, was collected as the cell-free extract [11].

#### 2.3.2. 2,2′-Azino-Bis (3-Ethylbezothiazoline)-6-Sulfonic Acid (ABTS) Radical Scavenging Ability

The ABTS radical scavenging assay was performed according to the method described by Farhat et al. (2022) [12]. Briefly, a 7 mmol/L ABTS solution and a 2.45 mmol/L K_2_S_2_O_8_ solution were mixed at a 1:1 ratio and allowed to react in the dark at room temperature for 12–16 h to generate the stock solution. It was then diluted with ethanol to achieve an absorbance of 0.70 ± 0.02 at 734 nm, which was used as the working solution. Next, 0.6 mL of the sample was added to a test tube containing 2.4 mL of the working solution. The mixture was incubated in the dark at room temperature for 6 min and measured at 734 nm. The ABTS radical scavenging activity was then calculated as:

(2) $$\mathrm{ABTS}\;\mathrm{radical}\;\mathrm{scavenging}\;\mathrm{ability}\;\left(\%\right)=\left[1-\frac{A_{1}}{A_{0}}\right]\times 100\%\;$$

A_0_: Sterile water was substituted for the absorbance of the sample; A_1_: the absorbance of the sample.

#### 2.3.3. Hydroxyl (OH^−^) Radical Scavenging Ability

The hydroxyl radical was measured according to the method described by Li et al. (2023) with slight modification [13]. In brief, 2 mL of the sample, 2 mL of a 9 mmol/L ferrous sulfate solution, 2 mL of a 9 mmol/L salicylic acid solution, and 2 mL of an 8.8 mmol/L hydrogen peroxide solution were added to a container and thoroughly mixed. The mixture was then incubated at 37 °C for 30 min and measured at 510 nm. The percentage of hydroxyl radical scavenging activity was calculated as:

(3) $${\mathrm{OH}}^{-}\;\mathrm{radical}\;\mathrm{scavenging}\;\mathrm{ability}\;\left(\%\right)=\left[A_{0}-\frac{A_{1}-A_{2}}{A_{0}}\right]\times 100\%\;$$

A_0_: Sterile water was substituted for the absorbance of the sample; A_1_: the absorbance of the sample; A_2_: the absorbance of the experimental group with sterile water instead of hydrogen peroxide.

#### 2.3.4. Superoxide Anion (O_2_^−^) Radical Scavenging Ability

The superoxide anion radical scavenging activity was assessed by the method of Rwubuzizi et al. (2023) with minor modifications [14]. A mixture of 0.1 mL of the sample, 2.8 mL of Tris-HCl buffer (0.05 M, pH 8.2), and 0.1 mL of pyrogallol (0.05 mol/L) was incubated in a water bath at 25 °C for 4 min in the dark. The reaction was then stopped by adding 1 mL of 8 mol/L HCl, and the absorbance was measured at 320 nm. The scavenging activity against the superoxide anion radical was defined as:

(4) $$O_{2}^{-}\;\mathrm{radical}\;\mathrm{scavenging}\;\mathrm{ability}\;\left(\%\right)=\left[A_{0}-\frac{A_{1}-A_{2}}{A_{0}}\right]\times 100\%\;$$

A_0_: Sterile water was substituted for the absorbance of the sample; A_1_: the absorbance of the sample; A_2_: the absorbance of the experimental group with sterile water instead of pyrogallol.

#### 2.3.5. DPPH Radical Scavenging Ability

Detailed determination methods were described in Section 2.2.1.

#### 2.3.6. Reducing Activity

The assay of reducing activity was similar to the method of Ding et al. (2017) [7]. To put it simply, 0.5 mL of the sample was mixed with 0.5 mL of 1% (*w*/*v*) potassium ferricyanide and 0.5 mL of 0.2 M phosphate-buffered saline (PBS, pH = 6.6). The mixture was incubated in a 50 °C water bath for 20 min. After cooling, 0.5 mL of 10% (*w*/*v*) trichloroacetic acid was added, and the solution was centrifuged at 4500 rpm for 10 min at 4 °C. Then, 1 mL of the supernatant was mixed with 1 mL of 0.1% (*w*/*v*) FeCl_3_ solution and 1 mL of sterile water. After standing at room temperature for 10 min, the absorbance was measured at 700 nm. Sterile saline was used as a control instead of the sample, and the difference in absorbance between the control and experimental groups was used to evaluate the reducing activity, calculated using the following formula:

(5) $$\mathrm{Reducing}\;\mathrm{activity}\;={\;A}_{1}-A_{0}$$

A_0_: The absorbance of the sterile water group; A_1_: the absorbance of the sample.

### 2.4. Safety Evaluation of Strains

#### 2.4.1. Hemolysis Assay

The selected strains were inoculated onto Columbia blood agar plates (Changde BKMAM Biotechnology Co., Ltd., Changde, China) and incubated at 37 °C for 48 h to observe the formation of hemolytic zones around the colonies. *Staphylococcus aureus* ATCC 12598 was used as a positive control [15].

#### 2.4.2. Antibiotic Susceptibility Test

The antibiotic susceptibility of the LAB was evaluated using the disk diffusion method with some modifications [16]. A 0.1 mL suspension of the tested LAB strains, adjusted to 10^8^ CFU/mL, was spread onto MRS agar plates. Antibiotic disks containing ampicillin (10 μg), chloramphenicol (30 μg), tetracycline (30 μg), penicillin (10 μg), gentamicin (10 μg), ciprofloxacin (5 μg), erythromycin (15 μg), ceftriaxone (30 μg), cotrimoxazole (25 μg), and lincomycin (2 μg) were aseptically placed on the agar surface and gently pressed to ensure proper adhesion. The diameter of the inhibition zones was measured after incubation at 37 °C for 24–48 h.

### 2.5. Growth Characteristic Evaluation

The LAB were inoculated into MRS broth at a 3% (*v*/*v*) ratio and incubated at 37 °C for 24 h. The OD_600_ nm value of the culture medium was measured every 2 h to construct the growth curve. In addition, the strains were incubated under temperature (4 °C, 25 °C, 37 °C, and 45 °C) and NaCl (3.0%, 5.0%, 8.0%, and 10%) stress behaviors for 24 h, and the growth curve of the strains was monitored and recorded [17].

### 2.6. Adhesion Evaluation

The adhesion capacity of the strain was assessed through measurements of hydrophobicity, auto-aggregation, and co-aggregation [18,19]. The LAB strains and pathogenic bacteria, including *Escherichia coli* O157, *Salmonella typhimurium* ATCC 14028, and *Staphylococcus aureus* ATCC 12598, were incubated overnight at 37 °C.

The bacterial suspension was mixed with 2 mL of the three organic solvents (xylene, chloroform, and ethyl acetate), swirled for 5 min, and left at room temperature for 1 h. The percentage of hydrophobicity was calculated as:

(6) $$\mathrm{Hydrophobicity}\;\left(\%\right)=\left[1-\frac{A_{t}}{A_{0}}\right]\times 100\%\;$$

A_0_: The absorbance of the sample; A_t_: the absorbance of the sample reacted with solvent after 1 h.

The bacterial suspension was incubated at 37 °C for 0, 2, 4, 6, and 24 h. At each period, the absorbance of the sample was measured and recorded at 600 nm. The auto-aggregation percentage was calculated as:

(7) $$\mathrm{Auto}-\mathrm{aggregation}\;\left(\%\right)=\left[1-\frac{A_{t}}{A_{0}}\right]\times 100\%\;$$

A_0_: The absorbance of the sample at 0 h; A_t_: the absorbance of the sample at 2, 4, 6, and 24 h.

The bacterial suspension was mixed with 2 mL of three different pathogenic organisms, stirred for 30 s, and incubated at 37 °C for 5 h. The co-aggregation percentage was calculated as follows:

(8) $$\mathrm{Co}-\mathrm{aggregation}\;\left(\%\right)=\left[1-\frac{2A_{s}}{A_{0}+A_{1}}\right]\times 100\%$$

A_0_: The absorbance of sample; A_1_: the absorbance of pathogenic bacteria; A_s_: the absorbance of the mixed suspension supernatant after 4 h.

### 2.7. Survival in the Simulated Gastrointestinal Tract

#### 2.7.1. Acid and Bile Salt Tolerance

The strains were inoculated into MRS broth, with the pH adjusted to 3.0, and incubated at 37 °C for 3 h. The absorbance at 600 nm was measured at the start (0 h) and after 3 h. Similarly, the strains were inoculated into MRS broth containing 0.3% bile salt (Solarbio, Beijing, China) and incubated at 37 °C for 24 h. The absorbance at 600 nm was measured at 0, 6, and 24 h. The survival rate was calculated by the following formula:

(9) $$\mathrm{Survival}\;\mathrm{rate}\;\left(\%\right)=\frac{A_{t}}{A_{0}}\times 100\%\;$$

A_0_: The absorbance of the sample at 0 h; A_t_: the absorbance of the sample at 3, 6, and 24 h.

#### 2.7.2. Simulated Gastroenteric Fluid Tolerance

Minor modifications were implemented as outlined by Wong et al. (2021) [20]. The 1 mL of bacterial suspension, adjusted to 10^9^ CFU/mL, was mixed with 9 mL of simulated gastric fluid and incubated at 37 °C under anaerobic conditions with shaking for 3 h. Samples were collected at 0 and 3 h for plate counting. Afterward, 1 mL of the bacterial suspension, which had been incubated in simulated gastric fluid for 3 h, was transferred into 9 mL of simulated intestinal fluid and incubated anaerobically at 37 °C for 8 h. After all plates were incubated at 37 °C for 48 h, colony counts were performed. The strain survival rate was calculated using the following formula:

(10) $$\mathrm{Survival}\;\mathrm{rate}\;\left(\%\right)=\frac{{\mathrm{lg}}^{N_{1}}}{{\mathrm{lg}}^{N_{2}}}\times 100\%\;$$

N_1_: Number of colonies after gastrointestinal simulation, in CFU/mL; N_2_: number of colonies before gastrointestinal simulation, in CFU/mL.

### 2.8. Whole-Genome Sequencing of the QL01

#### 2.8.1. DNA Extract and Genome Sequence

The QL01 cell sample was sent to Biomarker Biotechnology Co., Ltd., Beijing, China for whole-genome sequencing. Genomic DNA was isolated using the QIAamp DNA Micro Kit (Qiagen, Dusseldorf, Germany), and DNA integrity was assessed by 1% agarose gel electrophoresis. The isolated DNA was then sequenced using both the PacBio RS platform (Pacific Biosciences, Menlo Park, CA, USA) and the Illumina HiseaXten platform (Illumina, San Diego, CA, USA) [21].

#### 2.8.2. Genome Assembly and Functional Annotation

The filtered subreads were assembled using Canu (version 1.5) software, followed by genome circularization using Circlator (version 1.5.5). The prediction of coding genes, tRNA genes, rRNA genes, prophages, CRISPR sequences, genomic islands, secondary metabolite gene clusters, and promoters was performed using Prodigal (version 2.6.3), tRNAscan-SE (version 2.0.9), Infernal (version 1.1.3), PhiSpy (version 2.3), CRT (version 1.2), IslandPath-DIMOB (version 0.1), antiSMASH (version 5.0.0), and PromPredict (version 1). Functional annotation was conducted using Gene Ontology (GO) database, the Kyoto Encyclopedia of Genes and Genomes (KEGG) database, the Evolutionary Genealogy of Genes: Non-supervised Orthologous Groups (eggNOG, version 4.0), and Swiss-Prot database. Additionally, Carbohydrate-active enzymes (CAZy) database were utilized to predict the Virulence Factors of Pathogenic Bacteria (VFDB) database and the Comprehensive Antibiotic Research Database (CARD) database was employed for the analysis of pathogenicity and drug resistance analyses [22]. The versions, website links, and a brief description for these software and databases are summarized in Appendix A.

### 2.9. Statistical Analysis

All experiments were repeated in triplicate to ensure accuracy, and the results were expressed as mean ± standard deviation. Statistical analysis was performed using SPSS 22.0 for one-way analysis of variance (ANOVA), with a significance level set at *p* < 0.05. Histograms were generated using GraphPad Prism 9.

## 3. Results and Discussions

### 3.1. High-Throughput Screening of Antioxidant LAB

**Figure 1:**
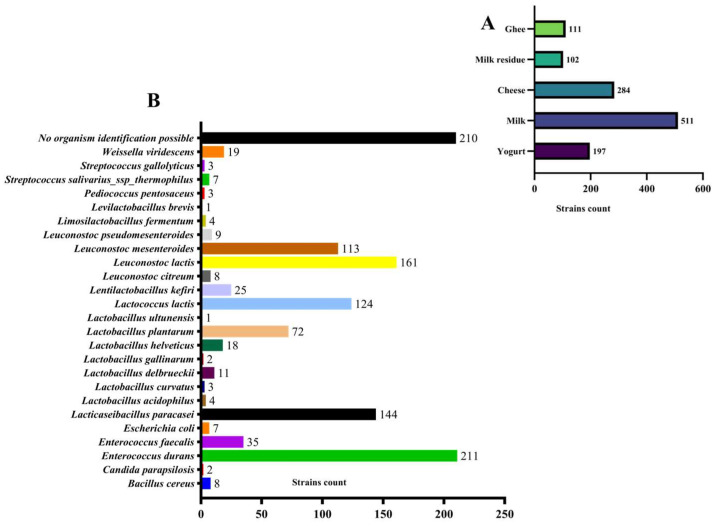
Statistical results of the isolation of strains. (**A**) Statistics on the number of isolates screened from different food samples in the Qinghai–Tibet Plateau. (**B**) Statistical results on the number of different species levels of isolates screened.

In recent years, there has been growing interest in screening for antioxidant probiotics. This chapter focused on the isolation of probiotic potential strains from yak milk and dairy products originating from the Qinghai–Tibet Plateau, with the goal of identifying those with strong antioxidant potential. As shown in Figure 1, a total of 1205 strains were isolated from yak milk and other samples. These strains were identified and revealed a high abundance of bacterial species in the collected samples, with *Enterococcus durans* being particularly prevalent, comprising 211 of the isolated strains.

**Figure 2:**
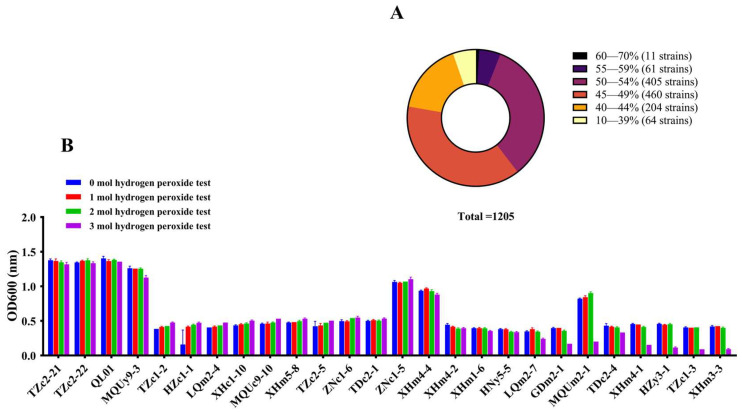
DPPH test and hydrogen peroxide tolerance test of isolated strains. (**A**) The distribution of the DPPH scavenging rate in the fermentation broth of 1205 strains, with different colors representing the ranges of DPPH scavenging ability. (**B**) Growth of the target 26 strains in the different concentrations of hydrogen peroxide.

Extensive efforts have been made to screen LAB for high antioxidant activity using in vitro whole-cell biochemical assays. For example, Li et al. (2023) [13] identified three strains with antioxidant potential, *Lactobacillus rhamnosus* S51, *Lactobacillus plantarum* S184, and *Lactobacillus fermentum* S7, using the DPPH radical scavenging assay from fermented food (water kefir). We screened strains with high antioxidant potential from a pool of 1205 isolates using the same method. As shown in Figure 2A, the DPPH radical scavenging rates of the strains isolated in this study ranged from 17.37% to 67.09%, with detailed data provided in Table S1. According to Sanzani et al. (2013) [23], LAB with a DPPH scavenging rate above 30% were generally considered to exhibit strong antioxidant activity. Based on this criterion, we identified 477 strains in our study that demonstrated excellent performance, with scavenging rates between 50% and 70%. Of these, 133 strains (with a clearance rate of ≥53%) were chosen for the hydrogen peroxide tolerance test.

Hydrogen peroxide is a by-product of normal oxygen metabolism, which can be effectively neutralized by the antioxidant system to maintain a dynamic balance between oxidation and antioxidation. Previous studies have demonstrated that LAB can tolerate certain concentrations of hydrogen peroxide and exhibit high antioxidant activity [24]. The result of the hydrogen peroxide tolerance test was presented in Table S2. Six strains were excluded from the analysis due to their loss of growth capacity during the experiment. All strains exhibited strong growth under 0 mmol/L hydrogen peroxide. However, the growth of the strains was increasingly affected as the concentration of hydrogen peroxide rose. When the concentration reached 3 mmol/L, most strains failed to grow normally, and only 50 strains demonstrated growth and tolerance to hydrogen peroxide. We selected 26 strains that demonstrated the best growth at 3 mmol/L for subsequent investigation, as illustrated in Figure 2B and Table 1. Interestingly, strain HZc1-1 demonstrated improved growth with increasing concentrations of hydrogen peroxide. Generally, the effect of hydrogen peroxide concentration on bacterial growth varies among strains. This phenomenon may occur because the concentration of hydrogen peroxide is relatively low for the HZc1-1, thereby enhancing its antioxidant capacity and adaptability, potentially promoting its growth.

LAB are known to exhibit species-specific differences in their free radical scavenging mechanisms [4]. These differences are primarily reflected in the distinct components of the bacteria responsible for antioxidant activity. For instance, the fermented supernatant of *Lactobacillus kefiri* demonstrated the highest free-radical scavenging capacity, while *Lactobacillus plantarum* showed the most powerful activity in its whole-cell form, as reported by Zhang et al. (2022) [25] and Lee et al. (2023) [26]. Moreover, cell-free extracts of various strains have also been shown to possess superior scavenging capacities [27]. To further investigate the components responsible for antioxidant activity in the 26 selected strains, we evaluated their antioxidant capacity using five distinct metrics: ABTS radical, hydroxyl radical, superoxide anion radical, DPPH radical, and reducing activity tests. The detailed results of these antioxidant tests, including the specific components analyzed, were provided in Table S3. A particularly striking observation was the significant variation in antioxidant behavior across different fractions of the same strain. Notably, the reducing activity was predominantly concentrated on the CFSs, with only a few strains displaying minimal reducing activity in the ICs, and no activity detected in the CFEs. This highlights the potential role of bioactive enzymes and non-enzymatic metabolites produced by probiotics, such as superoxide dismutase, glutathione reductase, glutathione, and melatonin, which are primarily found in the CFS fraction. Emerging evidence, including the study by Jin et al. (2024) [28], also suggested that these molecules play a crucial role in scavenging free radicals, and are produced in CFS fraction. Based on these findings, we selected nine strains with a strong antioxidant capacity (QL01, TZc1-2, ZNc1-5, XHm4-2, MQUm2-1, TDc2-4, XHm4-1, Hzy3-1, and XHm3-3) for further experimentation.

### 3.2. Safety Evaluation

#### 3.2.1. Hemolytic Activity

**Figure 3:**
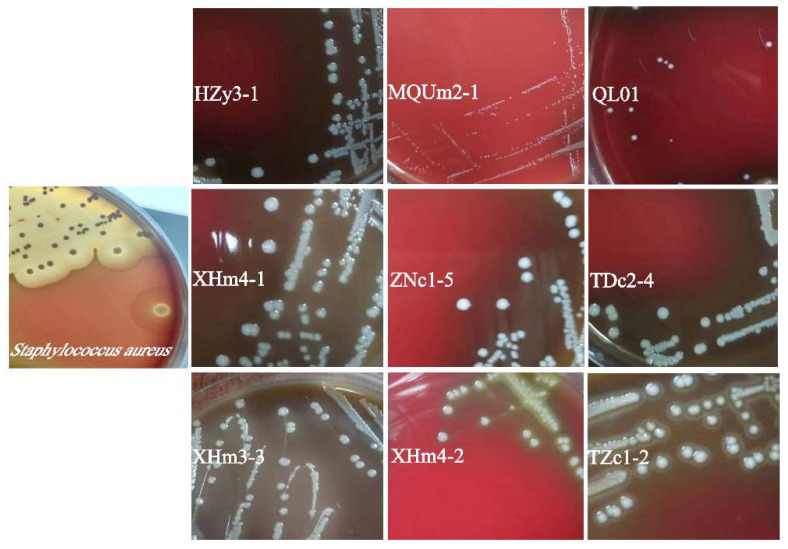
The hemolytic activity of the strain was analyzed, and the strain information is marked in white in the figure.

Hemolytic activity is an important indicator for evaluating the safety profile of probiotics [29]. As shown in Figure 3, two strains (TZc1-2 and XHm4-2) exhibited complete erythrocyte lysis, classifying them as beta-hemolytic, similar to the positive control strain. The remaining seven strains displayed no hemolytic activity, suggesting their safety for human use in terms of hemolytic potential. Therefore, these seven strains were selected for further investigation in the study.

#### 3.2.2. Antibiotic Susceptibility

Antibiotic sensitivity is a key factor in the safety evaluation of probiotics, as it plays a critical role in determining their suitability for human use. It is especially important to assess susceptibility to common antibiotics, given that those commercial probiotics are not allowed to carry and transfer antibiotic resistance genes in the human gut [30]. In this study, the antibiotic susceptibility of 7 LAB strains was tested against a panel of 10 antibiotics (Table 2). The results showed that all strains were sensitive to ampicillin, chloramphenicol, and penicillin. XHm4-1, QL01, ZNc1-5, and MQUm2-1 exhibited susceptibility to the majority of antibiotics. However, all strains except MQUm2-1 were resistant to gentamycin. Gentamycin is an aminoglycoside antibiotic that inhibits bacterial protein synthesis. Resistance to gentamycin has been observed in some LAB, aligning with our findings [31]. The overall sensitivity to most antibiotics supports the safety profile of these strains as potential probiotics [32].

### 3.3. Growth Characteristics

**Figure 4:**
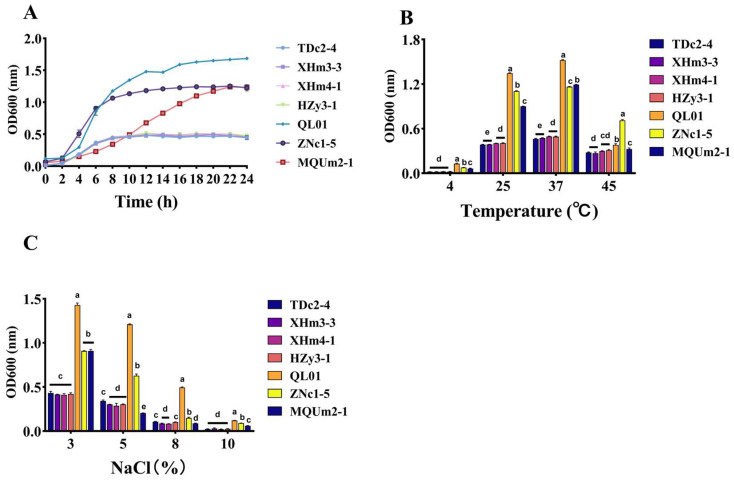
Growth characteristics of LAB. (**A**) Growth curves. (**B**) Effects of different temperatures (4, 25, 37, and 45 °C) on the growth of strains. (**C**) Effects of different sodium chloride concentrations (3, 5, 8, and 10%) on the growth of strains. (a–e) means that columns with other superscript letters differ per *p* < 0.05.

Probiotics encounter significant challenges related to survival due to the harsh conditions they face during processing, storage, and transport to the gastrointestinal tract, where they are ultimately digested. As a result, strains that demonstrate strong growth performance, tolerance to temperature fluctuations, and resistance to osmotic pressure are better suited for use as probiotics [34,35]. The growth performance of these strains can be visually assessed through growth curves. Figure 4A illustrates the growth curves of seven LAB strains cultured in MRS broth over a 24 h period. The concentration of all strains increased to varying extents as time progressed. All strains entered the exponential growth phase approximately 2 h after inoculation. By 8 h, strains TDc2-4, XHm3-3, XHm4-1, and Hzy3-1 had transitioned to the stationary phase, exhibiting relatively low growth and multiplication rates. Among the strains, QL01 exhibited the most boomed growth after reaching the stationary phase. The behavior is influenced by a variety of factors, including cellular by-products, nutrient availability, pH, temperature, and substrate concentration [36].

The effects of different temperatures on the strains were shown in Figure 4B. The findings indicated a pattern of initial increase followed by a decrease in the OD_600_ of each strain with rising culture temperature, exhibiting rapid growth at 25 °C and reaching peak values at 37 °C. Notably, the OD_600_ of TDc2-4, XHm3-3, XHm4-1, and Hzy3-1 remained consistently low across all temperature conditions, with QL01 exhibiting the highest OD_600_ at 4, 25, and 37 °C.

The stress test with different concentrations of NaCl is shown in Figure 4C. The results demonstrated a gradual decline in OD_600_ values for the LAB strains as NaCl concentration increased. Among the four strains of *Enterococcus faecalis*, they displayed the lowest tolerance to NaCl, while QL01 exhibited the highest tolerance. Notably, QL01 exhibited significantly better growth than the other LAB strains at a NaCl concentration of 10%, allowing it to maintain a relative advantage under osmotic pressure, which could be beneficial in the harsh conditions of the gastrointestinal tract [37].

### 3.4. Adhesion Ability

**Figure 5:**
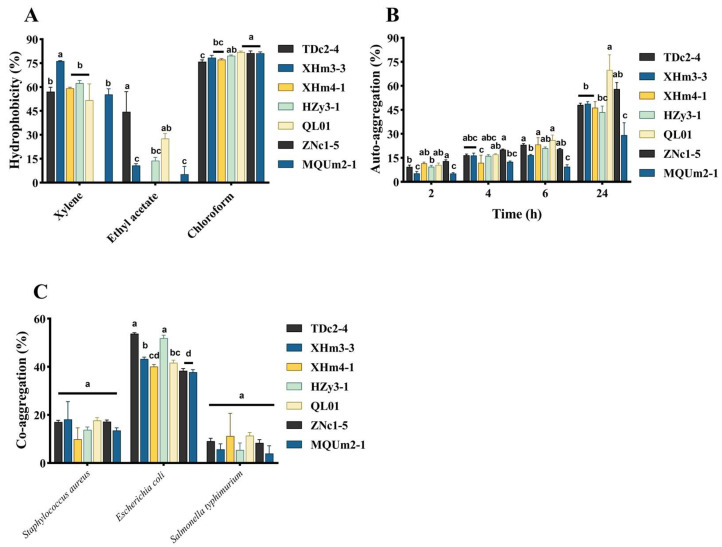
Adherence to LAB. (**A**) Hydrophobicity. (**B**) Auto-aggregation. (**C**) Co-aggregation. (a–d) means that columns with other superscript letters differ per *p* < 0.05.

Hydrophobicity is determined by the presence of hydrophobic components in the outer membrane of microorganisms, which enhances bacterial adhesion to intestinal epithelial cells [38]. In the phase separation behavior between organic hydrocarbons and water, the strains undergo hydrophobic partitioning. The hydrophobicity of the cell surface can be assessed by measuring the change in the number of strains in the aqueous phase [39]. All strains exhibited the highest hydrophobicity toward the acidic solvent chloroform (ranging from 75.97% to 82.00%), followed by the polar solvent xylene (51.78% to 76.28%), with the lowest hydrophobicity observed toward ethyl acetate (5.30% to 44.53%). Notably, XHm3-3 showed the highest hydrophobicity toward xylene (76.28%), while TDc2-4 exhibited the greatest hydrophobicity toward ethyl acetate (44.53%). ZNc1-5 and XHm4-1 demonstrated minimal hydrophobicity toward xylene and ethyl acetate, respectively (Figure 5A). These results indicated considerable variation in the hydrophobicity of different strains toward specific hydrocarbon solvents. Consistent with our findings, Pelletier et al. (1997) [40] reported that *Lactobacillus rhamnosus* displayed higher surface hydrophobicity toward chloroform compared to ethyl acetate, with hydrophobicity toward ethyl acetate ranging only from 11.7% to 16.5%. Similarly, Das et al. (2016) [41] observed significant differences in hydrophobicity among three LAB isolates of marine origin. This emphasized the variability in surface hydrophobicity among different bacterial strains.

In general, LAB with higher self-aggregating abilities tend to adhere more strongly to intestinal epithelial cells, thereby forming a barrier that effectively inhibits the colonization and invasion of pathogenic bacteria [42]. Figure 5B illustrated the auto-aggregation of seven strains at 2, 4, 6, and 24 h. The auto-aggregation rates were time-dependent, peaking at 24 h. They were associated with physiological activities and metabolite production during the culture phase, both of which enhance the auto-aggregation capacity [43]. Notably, significant differences in auto-aggregation rates were observed among the strains. At 24 h, QL10 exhibited a significantly higher agglutination rate of 69%. Zhang et al. (2022) [44] reported a similar time-dependent behavior in the auto-agglutination of LAB, supporting our findings. In contrast, Azat et al. (2016) [45] found that the *Lactobacillus rhamnosus* R4 displayed an auto-aggregation rate of only 45.83% after 24 h of incubation at 37 °C, which was considerably lower than that of the QL10 identified in our study.

The co-aggregation capacity of probiotics with pathogenic bacteria has been documented to establish a protective barrier against pathogen adhesion and colonization [46]. In our study, all strains exhibited the strongest co-aggregation ability with *Escherichia coli* (37.78–53.79%), with TDc2-4 and Hzy3-1 showing notably higher and statistically significant co-aggregation rates. In contrast, the co-aggregation abilities of all strains with *Staphylococcus aureus* (9.92–18.13%) and *Salmonella typhimurium* (3.96–11.37%) were relatively low. Although QL01 demonstrated a higher co-aggregation ability than the other strains, no significant differences were observed between QL01 and the others (Figure 5C). Our findings indicated that different strains possess varying abilities to co-aggregate with pathogenic bacteria, with QL01 showing potential in preventing the colonization of most pathogenic bacteria in the intestine. These results were consistent with prior research, which suggested that the co-aggregation characteristics of LAB were linked to the composition and structure of their surfaces [47].

### 3.5. Growth Under Simulated Gastrointestinal Environment

#### 3.5.1. Tolerance of LAB to Acid and Bile Salts

**Figure 6:**
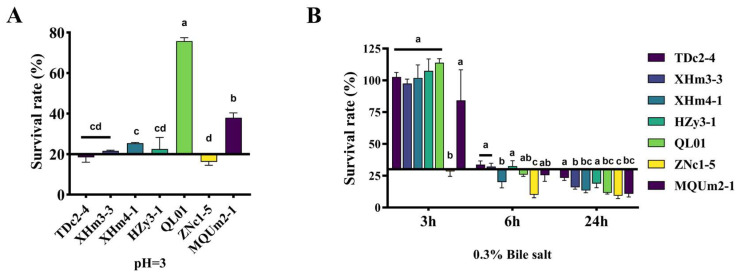
Tolerance of LAB to acid and bile salts. (**A**) Three-hour survival rate of pH = 3. (**B**) Three-hour, six-hour, and twenty-four-hour survival rates of 0.3% bile salt. (a–d) means that columns with other superscript letters differ per *p* < 0.05.

One of the essential properties of probiotics is the ability to survive in the harsh, low-pH environment of gastric acid. Probiotics need to withstand a survival period of 1.5 to 2 h in conditions with a pH between 2 and 3 to exert beneficial effects on host health [48]. Bile salt concentrations typically range from 0.03% to 0.30% in the small intestine, and probiotics need to exhibit tolerance to these bile salts for colonizing and regulating the gut microbiota effectively [49]. Therefore, it is a crucial characteristic of high-quality probiotics for strong resistance to both acidic environments and bile salts. All tested strains survived 3 h at a pH of 3, though significant variation in acid tolerance was observed. Figure 6A indicated that QL01 exhibited the highest survival rate (76%), while the remaining strains only had survival rates below 40%; thus it can be seen that QL01 has superior acid tolerance.

The results of 0.3% bile salt tolerance experiments are shown in Figure 6B. QL01 still had the highest survival rate after 3 h of incubation, and other strains also demonstrated strong survival, except for ZNc1-5. However, ZNc1-5 continued to exhibit the lowest tolerance and TDc2-4 showed the highest survival rate at 6 and 24 h. Consistent with prior research, the survival of *Lactobacilli* under acidic and bile salt conditions was found to be time-dependent [50].

#### 3.5.2. Tolerance of LAB to Simulated Gastrointestinal Conditions

In addition to the acidic environment, digestive enzymes present in gastrointestinal fluids, such as pepsin and pancreatic enzymes, also exert a strong inhibitory effect on LAB [51]. Therefore, this study evaluated the tolerance of strains to simulated gastrointestinal fluids. The results demonstrated that all strains achieved survival rates above 90%, apart from TDc2-4 and XHm3-3 (Table 3). However, only QL01 maintained a survival rate exceeding 80% with viable counts surpassing the critical threshold of 10^6^ CFU/mL after 8 h of incubation in simulated intestinal fluids. A level of 10^6^ CFU/mL is necessary for retaining the functional properties of the bacterial cells [52].

In conclusion, *Lactobacillus plantarum* QL01 exhibited excellent probiotic properties, including boomed growth across the stress test for temperature and NaCl, strong adhesion capabilities, and high survival rates under simulated gastrointestinal conditions. Based on these findings, QL01 was selected for whole-genome sequencing to further investigate its safety profile and physiological characteristics.

### 3.6. Complete Genome Sequencing

#### 3.6.1. Genome Features

**Figure 7:**
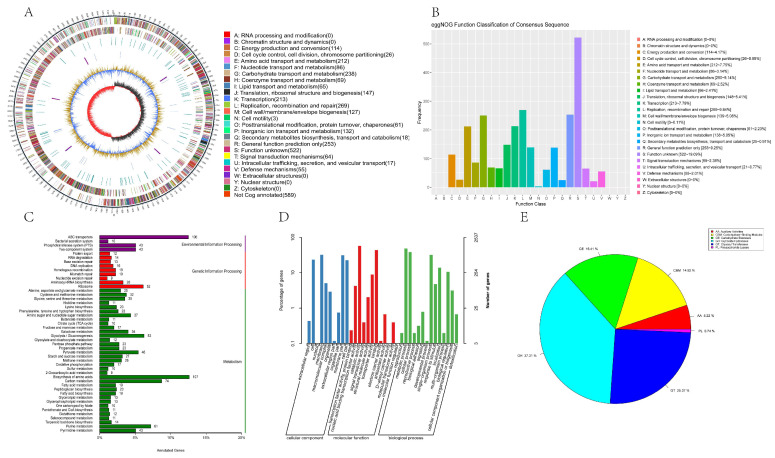
*L. plantarum* QL01 circular genome and its function annotation. (**A**) Map of the genome circle. The outermost circle is the genome size marker. The second and third circles are genes on the plus strand and minus strand of the genome, respectively. Different colors represent different eggNOG functional classes. The fourth loop was a repeat sequence. The fifth circle is tRNA (blue) and rRNA (purple). The sixth circle is the GC content. The light-yellow part indicates that the GC content of this region is higher than the average GC content of the whole genome, whereas the blue part indicates the opposite. In addition, the higher the peak value is, the more significant the difference from the average GC content is. The innermost circle is GC-skew. The red part indicates that the G content in this area is lower than that of C, and the dark gray part indicates the opposite. (**B**) Evolutionary Genealogy of Genes: Non-supervised Orthologous Groups (eggNOG) functional classification. (**C**) Gene ontology (GO) analysis. (**D**) Kyoto Encyclopedia of Genes and Genomes (KEGG) pathways enrichment. (**E**) Carbohydrate-active enzymes (CAZy) analysis.

The genomic features of QL01 were found to closely resemble those of other documented strains [53]. The complete genome sequence of QL01 was a single circular chromosome of 3,404,517 bp with an average GC content of 44.38% (Figure 7A) and five plasmids. This genome contained 3280 coding sequences(CDS). Additionally, a total of 16 rRNA genes were identified, including six copies each of the 5S rRNA and five copies each of the 16S rRNA and 23S rRNA, along with 68 tRNA genes. The main genomic features are listed in Table 4.

#### 3.6.2. Genome Annotation

Using eggNOG annotation (Figure 7B), it was revealed that the QL01 genome contained a significant number of coding genes for proteins with unknown functions (S, 522), suggesting the strain’s potential to produce a diverse range of unique functional proteins. This observation aligned with previous research findings [54]. Subsequently, the QL01 genome displayed a notably high number of genes associated with replication, recombination, and repair (L, 269). Additionally, there were genes also involved in general function prediction (R, 253), carbohydrate transport and metabolism (G, 250), transcription (K, 213), and amino acid transport and metabolism (E, 250).

Based on the GO database, the gene functions were categorized into three main domains: cellular component, molecular function, and biological process. Analysis revealed that the highest number of gene annotations (3914) were associated with biological processes, particularly in areas related to metabolic processes, cellular processes, and single-organism processes. Additionally, 3017 genes were annotated under cellular components, with a focus on the cell membrane and cellular structures. These cellular component annotations suggested that QL01 possessed a strong ability to form biofilms, which could protect it from external environmental stressors [55]. Moreover, 3012 genes were linked to molecular functions, primarily involving catalytic activity, binding sites, and transport activities (Figure 7C).

A total of 1490 genes were functionally annotated in the KEGG database (Figure 7D). Most of these genes were associated with metabolic pathways, particularly involved in amino acid biosynthesis, carbon metabolism, and purine metabolism. Our findings suggested that QL01 possessed a strong metabolic capacity and high adaptability to the environment [55]. Additionally, a subset of genes was linked to environmental information-processing functions, including 106 genes associated with ABC transporters. This indicated that QL01 might have a more complex secondary metabolite synthesis pathway and regulatory network [56].

#### 3.6.3. Carbohydrate-Active Enzymes

Analyzing with the CAZy database, 134 genes of the QL01 genome were divided into six CAZy classes (Figure 7E). Glycoside hydrolases (GHs) accounted for 37.31% of the total annotated genes in QL01, followed by glycosyltransferases (GTs) at 25.37%, carbohydrate esterases (CEs) at 16.41%, carbohydrate binding modules (CBMs) at 14.92%, auxiliary activities (AAs) at 5.22%, and polysaccharide lyases (PLs) represented only 0.74%. GHs were the predominant enzyme class in QL01, and these enzymes played a crucial role in the hydrolysis of complex carbohydrates and were widely recognized as pivotal components of intestinal flora’s carbohydrate metabolism [57]. Furthermore, the presence of GTs suggested that QL01 might possess significant probiotic potential in combating pathogens and eliciting immune responses [58].

#### 3.6.4. Annotation of Drug Resistance Genes and Virulence Factors

The CARD annotated a resistance gene poxtA, which encodes a ribosomal protection protein of the ATP-binding cassette (ABC-F) family and can lead to reduced drug sensitivity or produced resistance to oxazolidinone, chloramphenicol, and tetracycline antibiotics [59]. Despite the presence of this resistance gene, in vitro experiments have demonstrated that QL01 did not display resistance to chloramphenicol and tetracycline drugs, indicating a complex relationship between genotype and phenotype. Chloramphenicol primarily exerts its effects by inhibiting protein synthesis in bacteria. LAB depend on comparable translational mechanisms for intracellular protein synthesis, making them susceptible to chloramphenicol [24].

A total of 17 genes were recorded in the VFDB annotation results (Table S4). Although many potential virulence factor genes were detected in the QL01 genome, most of these genes showed less than 50% similarity. The low nucleotide homology suggested that the evolutionary relationship between these genes was distant, which might result in their lack of expression or significant pathogenic effects. Therefore, they could not be classified as virulently expressed genes [60]. According to the KEGG database, these genes were predominantly associated with carbohydrate metabolism, amino acid metabolism, nucleotide metabolism, and lipid metabolism. Therefore, it remained uncertain whether these virulence factor genes were responsible for producing harmful metabolites [61]. Furthermore, QL01 did not detect the presence of aggressive virulence factors commonly associated with pathogenic bacteria, including gelatinase (gelE), hyaluronidase (hyl), enterococcal surface protein (esp), cytolysin (cylA), endocarditis antigen (efaA), collagen adhesion (ace), hemolysin (hbl), and cytotoxin K (cytK), as well as non-hemolytic enterotoxin [62].

#### 3.6.5. Stress-Related Genes in QL01

Table 5 shows stress-related genes of QL01, including 4 universal stress proteins, 11 proteases and chaperones, 5 heat-shock stress proteins, 2 cold-shock stress proteins, 24 acid stress response genes, 2 alkaline stress response genes, 17 bile salt stress response genes, 15 adhesion-related genes, and 38 oxidative stress response genes. The QL01 contained heat-shock stress proteins and cold-shock stress proteins, the expression of which could enhance the ability to withstand both low and high temperatures, consistent with our in vitro experimental findings. In addition, gene function analysis also identified 15 genes associated with adhesion, providing insight into its excellent adhesion capability.

In addition to stress response genes related to temperature, acid-base, and adhesion, the most extensively annotated genes in the QL01 genome were associated with oxidative stress, including nicotinamide adenine dinucleotide (NADH) peroxidase, glutathione peroxidase, glutathione reductase, thioredoxin, sulfoxide reductase, and thioredoxin reductase genes. Catalase and NADH oxidase/peroxidase are directly involved in the detoxification of hydrogen peroxide and ROS. Glutathione reductase is an important antioxidant enzyme that is responsible for maintaining glutathione, which is one of the main antioxidant metabolites [63]. The presence of these genes in QL01 enabled it to withstand oxidative stress, which was consistent with the antioxidant capacity demonstrated in in vitro experiments. The presence of these genes has significantly enhanced the relative adaptability and tolerance of QL01 to challenging environmental conditions.

## 4. Conclusions

In this study, 9 strains of LAB with strong antioxidant potential were comprehensively screened from a collection of 1205 strains derived from yak milk and dairy products in the Qinghai–Tibet Plateau using various antioxidant assays. Following in vitro safety evaluations and the analysis of probiotic characteristics, QL01 was identified as a safe strain, exhibiting favorable growth characteristics, strong adhesion ability, and resilience to acidic conditions, bile salts, and gastrointestinal fluids. Genomic analysis further confirmed QL01’s safety profile and revealed the presence of genes linked to probiotic functions, adaptive responses, and oxidative stress resistance. These genes were associated with amino acid metabolism, carbohydrate-active enzymes, stress responses, and adhesion to intestinal epithelial cells. QL01 was conserved in the China center for type culture collection under the number CCTCC NO: M 20241615. The findings from this study offer a valuable reference for the screening of LAB with antioxidant properties. Future research will focus on in vivo experiments using animal models to further elucidate the antioxidant mechanisms of QL01 and explore its potential applications in the development of functional foods, pharmaceuticals, and cosmetics with antioxidant benefits.

## Figures and Tables

**Table 1:** Detailed information on the 26 lactic acid bacteria isolates.

	Strains	Microorganisms	Origin		Strains	Microorganisms	Origin
1	TZc2-22	*Lactobacillus plantarum*	Ghee	14	ZNc1-5	*Pediococcus pentose*	Ghee
2	TZc2-21	*Lactobacillus plantarum*	Ghee	15	XHm4-4	*Weissella viridescens*	Milk
3	QL01	*Lactobacillus plantarum*	Milk	16	XHm4-2	*Leuconostoc lactis*	Milk
4	MQUy9-3	*Lactobacillus plantarum*	Yogurt	17	XHm1-6	*Leuconostoc lactis*	Milk
5	TZc1-2	*Enterococcus durans*	Ghee	18	HNy5-5	*Leuconostoc lactis*	Yogurt
6	HZc1-1	*Enterococcus durans*	Cheese	19	LQm2-7	*Leuconostoc lactis*	Milk
7	LQm2-4	*Enterococcus durans*	Milk	20	GDm2-1	*Leuconostoc lactis*	Milk
8	XHEc1-10	*Enterococcus durans*	Cheese	21	MQUm2-1	*Levilactobacillus brevis*	Milk
9	MQUc9-10	*Enterococcus durans*	Milk residue	22	TDc2-4	*Enterococcus faecalis*	Cheese
10	XHm5-8	*Enterococcus durans*	Milk	23	XHm4-1	*Enterococcus faecalis*	Milk
11	TZc2-5	*Enterococcus durans*	Ghee	24	HZy3-1	*Enterococcus faecalis*	Yogurt
12	ZNc1-6	*Enterococcus durans*	Ghee	25	TZc1-3	*Enterococcus faecalis*	Ghee
13	TDc2-1	*Enterococcus durans*	Cheese	26	XHm3-3	*Enterococcus faecalis*	Milk

**Table 2:** Antibiotic susceptibility of the LAB strains.

Antibiotics	Judgment Standard of Bacteriostatic Circle Diameter (mm)			TDc2-4	XHm3-3	XHm4-1	HZy3-1	QL01	ZNc1-5	MQUm2-1
	R	I	S							
Ampicillin	≤16	-	≥17	S	S	S	S	S	S	S
Chloramphenicol	≤12	13–17	≥18	S	S	S	S	S	S	S
Tetracycline	≤14	15-18	≥19	R	R	I	R	S	I	S
Penicillin	≤14	-	≥15	S	S	S	S	S	S	S
Gentamycin	≤13	13–14	≥15	R	R	R	R	R	R	S
Ciprofloxacin	≤15	16–20	≥21	R	I	R	R	R	R	R
Erythromycin	≤13	14–22	≥23	R	S	S	S	S	S	S
Ceftriaxone	≤13	13–21	≥21	S	S	S	I	S	S	I
Cotrimoxazole	≤10	10–12	≥12	R	R	S	S	R	R	R
Lincomycin	≤14	14–21	≥21	R	R	R	R	S	I	S

Note: resistant according to the guidelines of the Institute of Clinical and Laboratory Standards Institute (CLSI) [33]; S: susceptible; I: intermediate; R: resistant.

**Table 3:** Gastric and intestinal fluid tolerance of LAB.

Strains	Simulated Gastric Juice			Simulated Intestinal Juice	
	0 h (log CFU/mL)	3 h (log CFU/mL)	Survival Rate (%)	8 h (log CFU/mL)	Survival Rate (%)
TDc2-4	8.72 ± 0.05 ^ab^	6.45 ± 0.20 ^e^	74.02 ± 2.42 ^d^	-	-
XHm3-3	8.75 ± 0.11 ^a^	7.14 ± 0.03 ^d^	81.57 ± 1.18 ^c^	-	-
XHm4-1	8.63 ± 0.06 ^ab^	8.23 ± 0.05 ^ab^	95.37 ± 1.05 ^a^	-	-
HZy3-1	8.69 ± 0.07 ^ab^	7.94 ± 0.11 ^c^	91.42 ± 0.74 ^b^	-	-
QL01	8.22 ± 0.04 ^d^	8.01 ± 0.03 ^bc^	97.47 ± 0.88 ^a^	6.52 ± 0.03 ^a^	81.42 ± 0.73 ^a^
ZNc1-5	8.36 ± 0.08 ^cd^	8.16 ± 0.06 ^bc^	97.60 ± 0.27 ^a^	2.80 ± 0.35 ^b^	34.30 ± 4.26 ^b^
MQUm2-1	8.54 ± 0.03 ^bc^	8.29 ± 0.01 ^a^	97.09 ± 0.28 ^a^	-	-

Note: ^a–e^ means within columns with different superscript letters are different per *p* < 0.05. “-” represents no survival.

**Table 4:** Features of the *L. plantarum* QL01 genome.

Attributes	Values
Genome size (bp)	3,404,517
Plasmids	5
GC content (%)	44.38
5S rRNA	6
16S rRNA	5
23S rRNA	5
tRNA	68
Total predicted CDSs	3280
Genomic island	19
Prophage	3
CRISPR number	12
Gene cluster	3
Promoter	4

**Table 5:** Stress-related genes of *L. plantarum* QL01.

Gene	Gene Symbol	Annotation
**Universal stress protein (4)**		
GE002013	SERP1273	Putative universal stress protein SERP1273
GE002237	SERP1273	Putative universal stress protein SERP1273
GE002316	SERP1273	Putative universal stress protein SERP1273
GE002427	SH1215	Putative universal stress protein SH1215
**Proteases and chaperones (11)**		
GE000509	clpP1	ATP-dependent Clp protease proteolytic subunit 1
GE000563	clpP1	ATP-dependent Clp protease proteolytic subunit 1
GE000711	clpP	ATP-dependent Clp protease proteolytic subunit
GE001110	clpE	ATP-dependent Clp protease ATP-binding subunit ClpE
GE001822	clpX	ATP-dependent Clp protease ATP-binding subunit ClpX
GE002972	clpL	ATP-dependent Clp protease ATP-binding subunit ClpL
GE003122	clpL	ATP-dependent Clp protease ATP-binding subunit ClpL
GE000904	clpC_6	ATP-dependent Clp protease, ATP-binding subunit ClpC
GE001561	hslU	ATP-dependent protease ATPase subunit HslU
GE000413	htpX	Protease HtpX homolog
GE000059	htrA	Serine protease Do-like HtrA
**Heat-shock stress (5)**		
GE000132	-	Heat shock protein HSP.16.4
GE002806	hspC2	Small heat shock protein C2
GE002254	-	18 kDa heat shock protein
GE002806	-	18 kDa heat shock protein
GE001682	hrcA	Heat-inducible transcription repressor HrcA
**Cold-shock stress (2)**		
GE000030	-	Cold-shock protein
GE000890	csp_2	Cold-shock protein
**Acid stress (24)**		
GE000187	nhaC	Na(+)/H(+) antiporter NhaC
GE002794	nhaC	Na(+)/H(+) antiporter NhaC
GE002031	atpC	ATP synthase epsilon chain
GE002032	atpD	ATP synthase subunit beta
GE002033	atpG	ATP synthase gamma chain
GE002034	atpA	ATP synthase subunit alpha
GE002035	atpH	ATP synthase subunit delta
GE002036	atpF	ATP synthase subunit b
GE002037	atpE	ATP synthase subunit c
GE002038	atpB	ATP synthase subunit a
GE000329	ldh	L-lactate dehydrogenase
GE000438	ldh1	L-lactate dehydrogenase 1
GE000790	ldhD	D-lactate dehydrogenase
GE000968	ldh2	L-lactate dehydrogenase 2
GE001709	ldhD	D-lactate dehydrogenase
GE001848	ldh	L-lactate dehydrogenase
GE000704	argH	Argininosuccinate lyase
GE000703	argG	Argininosuccinate synthase
GE000427	argC2	N-acetyl-gamma-glutamyl-phosphate reductase 2
GE002267	dapA	4-hydroxy-tetrahydrodipicolinate synthase
GE001952	dapH	2,3,4,5-tetrahydropyridine-2,6-dicarboxylate N-acetyltransferase
GE002848	gadB	Glutamate decarboxylase
GE000014	proB	Glutamate 5-kinase
GE000431	argF	Ornithine carbamoyltransferase
**Alkaline stress (2)**		
GE000832	asp23	Alkaline shock protein 23
GE000833	--	Alkaline shock protein 23
**Bile salt stress (17)**		
GE001263	SpyM3_0208	Probable ABC transporter ATP-binding protein SpyM3_0208
GE001583	yfmR	Uncharacterized ABC transporter ATP-binding protein YfmR
GE002054	BQ2027_MB1303C	Fatty acid ABC transporter ATP-binding/permease protein
GE000670	pstS1	Phosphate-binding protein PstS 1
GE000673	pstB1	Phosphate import ATP-binding protein PstB 1
GE000674	pstB2	Phosphate import ATP-binding protein PstB 2
GE000660	pstS	Phosphate-binding protein PstS
GE000886	ycnB	Uncharacterized MFS-type transporter YcnB
GE001433	SACE_5813	Uncharacterized MFS-type transporter SACE_5813
GE002389	ydeR	Uncharacterized MFS-type transporter YdeR
GE002391	ycnB	Uncharacterized MFS-type transporter YcnB
GE002439	yhcA	Uncharacterized MFS-type transporter YhcA
GE002770	ycnB	Uncharacterized MFS-type transporter YcnB
GE003020	yhcA	Uncharacterized MFS-type transporter YhcA
GE003093	yhcA	Uncharacterized MFS-type transporter YhcA
GE000080	cbh	Choloylglycine hydrolase
GE002272	-	Choloylglycine hydrolase
GE001437	cfa	Cyclopropane-fatty-acyl-phospholipid synthase
GE002556	yhaA	Putative amidohydrolase YhaA
**Adhesion ability (15)**		
GE001598	scpB	Segregation and condensation protein B
GE001599	scpA	Segregation and condensation protein A
GE000702	luxS	S-ribosylhomocysteine lyase
GE000717	eno1	Enolase 1
GE001623	eno2	Enolase 2
GE000715	pgk	Phosphoglycerate kinase
GE001706	tsf	Elongation factor Ts
GE000017	oppA	Oligopeptide-binding protein OppA
GE000434	oppA	Periplasmic oligopeptide-binding protein
GE000709	oppA	Oligopeptide-binding protein OppA
GE002739	aliB	Oligopeptide-binding protein AliB
GE001591	rps1	30S ribosomal protein S1
GE000810	-	Molecular chaperone GroEL
GE001679	dnaJ	Molecular chaperone DnaJ
GE000714	gap	Glyceraldehyde-3-phosphate dehydrogenase
GE001506	lspA	Lipoprotein signal peptidase
**Oxidative stress (38)**		
GE000223	trxA	Thioredoxin
GE000691	trxB	Thioredoxin reductase
GE001958	trxA	Thioredoxin
GE002867	trxA	Thioredoxin
GE001998	tpx	Thiol peroxidase
GE003056	-	Thioredoxin
GE003158	tpx	Thiol peroxidase
GE001165	msrA1	Peptide methionine sulfoxide reductase MsrA 1
GE001553	msrA	Peptide methionine sulfoxide reductase MsrA
GE001554	msrB	Peptide methionine sulfoxide reductase MsrB
GE001655	msrA	Peptide methionine sulfoxide reductase MsrA
GE000015	proA	Gamma-glutamyl phosphate reductase
GE002353	panE	2-dehydropantoate 2-reductase
GE001300	gnd	6-phosphogluconate dehydrogenase, decarboxylating
GE000074	fabG	3-oxoacyl-[acyl-carrier-protein] reductase
GE001419	fabG	3-oxoacyl-[acyl-carrier-protein] reductase FabG
GE002191	asd	Aspartate-semialdehyde dehydrogenase
GE002468	nrdD	Anaerobic ribonucleoside-triphosphate reductase
GE002467	nrdG	Anaerobic ribonucleoside-triphosphate reductase-activating protein
GE000856	ifcA	Fumarate reductase flavoprotein subunit
GE000985	SO_0970	Fumarate reductase flavoprotein subunit
GE000626	nrdF2	Ribonucleoside-diphosphate reductase subunit beta nrdF2
GE000627	nrdE1	Ribonucleoside-diphosphate reductase subunit alpha 1
GE001094	gor	Glutathione reductase
GE002730	gor	Glutathione reductase
GE000347	gshR1	Glutathione reductase
GE001094	merA	Glutathione reductase
GE001546	-	Glutathione reductase
GE002730	pdhD_1	Glutathione reductase
GE001243	npr	NADH peroxidase
GE002167	npr	NADH peroxidase
GE000212	gpo	Glutathione peroxidase
GE000073	arsC	Arsenate reductase
GE000756	arsC_1	Arsenate reductase
GE002800	-	Arsenate reductase
GE000691	trxB	Thioredoxin reductase
GE001546	pdhD	Dihydrolipoyl dehydrogenase
GE001849	pdhD	Dihydrolipoyl dehydrogenase

## Data Availability

Data will be made available on request.

## Supplementary Materials

The following supporting information can be downloaded at: https://www.mdpi.com/article/10.3390/antiox14020173/s1, Table S1: DPPH radical scavenging rate of 1205 lactic acid bacteria; Table S2: Growth of lactic acid bacteria at different concentrations of hydrogen peroxide; Table S3: Results of the determination of 5 antioxidant indexes in 26 lactic acid bacteria; Table S4: Putative virulence factors in the *Lactobacillus plantarum* QL01 genome.

## Appendix A

**Table A1:** Supplementary information regarding the software utilized for genomic feature analysis.

Software	Link	Brief Description
Canu v1.5	https://github.com/marbl/canu	Prepare the sequencing data, typically in a FASTQ format from Illumina or PacBio. Then, configure the parameters and execute Canu via the command line. Canu will generate log files in the designated output directory, which facilitates the monitoring of the workflow’s progress and enables the review of the results and processing details.
Circlator v1.5.5	https://github.com/sanger-pathogens/circlator	Prepare the sequencing data, then run Circlator to perform circularization and adjust the starting point. The tool will output the circularized assembly sequences along with detailed report files.
Prodigal v2.6.3	https://github.com/hyattpd/Prodigal	Prepare the genomic sequences for annotation, then run Prodigal, which employs a dynamic programming algorithm to predict genes in newly sequenced genomes, achieving high accuracy.
tRNAscan-SE v2.0.9	https://github.com/UCSC-LoweLab/tRNAscan-SE	The software identifies tRNAs in the genome by leveraging the characteristics of tRNA sequences and secondary structures, as well as associated promoter features, resulting in highly accurate predictions of tRNAs within the genome.
Infernal v1.1.3	http://eddylab.org/infernal/	According to the covariance model, the three classes of rRNAs in the genome can be predicted with high accuracy.
PhiSpy v2.3	https://sourceforge.net/projects/phispy/files/	The software predicts prophages by analyzing various characteristics of prophage DNA regions, including protein length, transcriptional strand orientation, AT skew, GC skew, and the presence of phage-related terminology in functional annotations. By integrating these features, the software can accurately predict prophage sequences within the genome.
CRT v1.2	http://www.room220.com/crt/	Prepare the genomic sequences for analysis, then run CRT to predict CRISPR elements within the genome.
IslandPath-DIMOB v0.1	https://github.com/brinkmanlab/islandpath	The software predicts gene islands in the genome based on the principle of using dinucleotide bias and the presence of at least one mobile gene.
antiSMASH v5.0.0	https://antismash.secondarymetabolites.org/#!/start	The software utilizes an implicit hidden Markov model tailored to specific types of gene clusters, allowing for the accurate identification of gene clusters that encode secondary metabolites across all known major chemical categories.
PromPredict v1	https://dna.mbu.iisc.ac.in/prompredict/prompredict.html	This software analyzes genomic DNA with varying GC contents and serves as a universal standard for predicting promoter regions in microbial genomes. By predicting both the sense and antisense strands of the genome, promoters located within 500 bp upstream of the gene with a prediction confidence level exceeding level 2 are selected as the predicted results.
GO releases20180910	http://geneontology.org/docs/download-ontology/	The workflow of the GO database encompasses data collection, term construction, annotation assignment, periodic updates, quality control, and ultimately, providing accessible data to users through a website and an API.
KEGG kegg_201703	https://www.genome.jp/kegg/	The workflow of the KEGG database includes data collection, pathway construction, annotation assignment, periodic updates, and providing accessible data and tools to users through a website and an API to support genomic analysis and metabolic research.
eggNOG v4.0	http://eggnog45.embl.de/	The workflow of the eggNOG database includes data collection, grouping of functional orthologs, annotation assignment, regular updates, and providing researchers with accessible data and tools to support functional genomics and evolutionary biology research through a user-friendly interface and API.
Swissprot swissprot-2019-07-31	https://www.uniprot.org/help/downloads	The workflow of the Swiss-Prot database includes data collection, annotation, quality control, regular updates, and interfaces that provide access to users to support bioinformatics studies and protein analysis.
CAZy	https://www.cazy.org/	The workflow of the CAZy database includes data collection, classification, annotation, regular updates, and providing users with accessible data and tools through the website and API to support functional studies and biotechnology applications of carbohydrate enzymes.
VFDB VFDB_20190423	http://www.mgc.ac.cn/VFs/	The workflow of the VFDB database includes data collection, annotation, alignment analysis, regular updates, and providing researchers with accessible data and tools through a website and API to support virulence factor studies of pathogenic microorganisms and investigations in related fields.
CARD CARD_20190423	https://card.mcmaster.ca/	The workflow of CARD includes data collection, annotation, identification and classification, regular updates, and provides researchers with accessible data and tools to support the analysis of antibiotic resistance research and its public health impact through a user-friendly interface and API.
