# Supplementary material for: Screening and Whole-Genome Analysis of Probiotic Lactic Acid Bacteria with Potential Antioxidants from Yak Milk and Dairy Products in the Qinghai–Tibet Plateau
Source: Antioxidants (Basel). 2025 Jan 31;14(2):173. doi: 10.3390/antiox14020173 (PMC11851503; doi:10.3390/antiox14020173)
Supplement: Supplementary file 1 [file antioxidants-14-00173-s001.zip › antioxidants-3433239-supplementary.pdf]

Table S1. DPPH radical scavenging rate of 1205 lactic acid bacteria.

	Strain	DPPH (%)		Strain	DPPH (%)		Strain	DPPH (%)		Strain	DPPH (%)
1	HZy1-1	51.84±1.73	303	MQUm2-14	52.21±2.56	605	TZc2-7	50.58±0.95	907	HNy5-8	46.14±0.44
2	HZy1-2	52.23±2.40	304	MQUc1-1	49.24±0.51	606	TZc2-8	60.00±0.71	908	HNy6-1	54.68±0.56
3	HZy1-3	52.79±1.54	305	MQUc1-2	43.21±2.43	607	TZc2-9	57.76±0.36	909	HNy6-2	45.49±2.32
4	HZy1-4	51.19±0.94	306	MQUc1-3	41.20±0.27	608	TZc2-10	52.11±2.06	910	HNy6-3	54.00±0.22
5	HZy1-5	51.59±1.06	307	MQUc1-4	48.32±5.10	609	TZc2-11	58.42±1.00	911	HNy6-4	48.29±2.02
6	HZy2-1	48.69±0.98	308	MQUc1-5	47.08±1.98	610	TZc2-13	57.58±0.25	912	HNy6-5	46.34±5.17
7	HZy2-2	47.76±0.27	309	MQUc1-6	45.86±1.79	611	TZc2-14	56.27±0.16	913	HNy6-6	51.17±0.43
8	HZy2-3	47.60±1.56	310	MQUc1-7	47.81±3.24	612	TZc2-15	56.20±1.73	914	HNc1-1	49.98±0.49
9	HZy2-4	47.75±1.06	311	MQUc1-8	40.48±4.07	613	TZc2-16	55.46±0.49	915	HNc1-2	49.26±1.26
10	HZy2-5	47.17±0.46	312	MQUc1-9	43.33±1.15	614	TZc2-17	53.88±0.54	916	HNc1-3	51.63±0.47
11	HZy2-6	48.31±0.52	313	MQUc1-10	47.44±1.31	615	TZc2-18	53.45±0.87	917	HNc1-4	48.52±1.01
12	HZy2-7	50.12±1.54	314	MQUc1-11	43.78±0.63	616	TZc2-19	54.23±0.38	918	HNc1-5	47.90±0.69
13	HZy2-8	50.37±1.14	315	MQUc1-12	40.17±2.86	617	TZc2-20	53.75±0.19	919	HNc1-6	52.55±0.51
14	HZy2-9	49.96±0.28	316	MQUc1-13	41.40±2.52	618	TZc2-21	53.73±1.04	920	HNc2-1	47.97±0.56
15	HZy2-10	50.37±1.07	317	MQUc1-14	39.99±1.94	619	TZc2-22	55.63±3.32	921	HNc2-2	43.88±0.63
16	HZy3-1	53.35±3.98	318	MQUc1-15	43.84±0.87	620	TZc2-23	54.63±1.07	922	HNc2-3	43.47±0.11
17	HZy3-2	49.78±0.30	319	MQUc1-16	39.69±0.52	621	TZc2-24	53.39±0.32	923	HNc2-4	45.06±2.56
18	HZy3-3	51.20±0.31	320	MQUc1-17	37.51±1.15	622	TZc2-25	52.68±0.91	924	ZKm1-1	44.18±1.91
19	HZy3-4	49.68±1.93	321	MQUc1-18	41.72±0.95	623	TZc2-26	53.47±0.33	925	ZKm1-2	45.62±2.03
20	HZy3-5	50.70±0.51	322	MQUc1-19	34.12±2.51	624	TZc2-27	48.85±0.04	926	ZKm1-3	43.48±0.40
21	HZy3-6	51.98±1.68	323	MQUc1-20	30.71±0.82	625	TZc2-28	49.13±0.17	927	ZKm1-4	46.83±1.49
22	HZy3-7	52.56±1.02	324	MQUc1-21	37.64±0.98	626	TZc2-29	49.89±0.58	928	ZKm1-5	44.05±0.35
23	HZc1-1	55.55±0.72	325	MQUc1-22	29.52±0.53	627	TZc2-30	49.47±0.07	929	ZKm1-6	43.11±1.30
24	HZc1-2	55.04±0.41	326	MQUc1-23	43.61±0.83	628	TZc2-31	51.70±0.11	930	ZKm1-7	45.41±0.18

25	HZc1-3	52.84±0.53	327	MQUc1-24	30.51±1.27	629	TZc2-32	51.93±0.30	931	ZKm2-1	43.59±2.28
26	HZc1-4	45.64±0.96	328	MQUc1-25	30.51±0.53	630	TZc2-33	48.75±0.18	932	ZKm3-1	44.07±0.78
27	HZc1-5	46.09±0.70	329	MQUc1-26	32.36±0.29	631	TZc2-34	49.19±0.37	933	ZKm3-2	43.86±0.29
28	HZc1-6	46.23±0.45	330	MQUc1-27	43.09±1.65	632	TZc2-35	50.23±0.45	934	ZKm3-3	42.96±1.03
29	HZc1-7	46.46±1.83	331	MQUc1-28	41.36±1.09	633	TZc2-36	48.93±0.14	935	ZKm3-4	47.47±0.83
30	HZc1-8	53.89±0.26	332	MQUc1-29	31.80±1.18	634	TZc2-37	48.80±1.03	936	ZKm3-5	45.75±0.78
31	HZc1-9	51.56±0.46	333	MQUc1-30	30.25±1.94	635	TZc3-1	51.37±0.21	937	ZKm3-6	42.58±0.43
32	HZc1-10	52.41±0.52	334	MQUc2-1	51.50±0.42	636	TZc3-2	48.87±2.18	938	ZKm3-7	44.74±0.71
33	HZc1-12	50.97±0.04	335	MQUc2-2	52.38±3.52	637	TZc3-3	49.91±0.67	939	ZKm3-8	44.59±0.49
34	HZc1-13	50.59±0.40	336	MQUc2-3	49.36±0.11	638	TZc3-4	44.15±0.66	940	ZKm3-9	44.66±0.69
35	HZc1-14	50.25±0.36	337	MQUc2-4	48.35±0.56	639	TZc3-5	47.78±0.62	941	ZKm3-10	45.09±0.47
36	HZc1-15	50.20±0.03	338	MQUc2-5	52.12±3.51	640	TZc3-6	51.64±1.11	942	ZKm4-1	41.19±0.50
37	HZc1-16	50.84±0.75	339	MQUc2-6	51.21±0.65	641	TZc3-7	48.23±1.06	943	ZKm4-2	40.28±0.40
38	HZc1-17	50.36±0.42	340	MQUc2-7	50.67±0.62	642	TZc3-8	48.22±0.66	944	ZKm4-3	39.73±0.07
39	HZc1-18	50.41±0.21	341	MQUc2-8	50.50±0.11	643	TZc3-9	47.58±0.74	945	ZKm4-4	39.78±1.76
40	HZc1-21	48.55±0.10	342	MQUc2-9	49.28±0.59	644	TZc3-10	48.63±0.77	946	ZKm4-5	42.81±1.04
41	HZc1-22	49.99±0.95	343	MQUc2-10	49.84±0.51	645	TZc3-11	50.37±0.65	947	ZKm4-6	43.97±0.89
42	HZc2-2	38.99±0.93	344	MQUc2-11	48.82±1.99	646	TZc3-12	47.57±0.77	948	ZKm4-7	40.53±0.10
43	HZc2-3	39.19±0.95	345	MQUc3-1	37.63±0.41	647	TZc3-13	48.58±2.95	949	ZKm5-1	44.15±2.36
44	HZc2-4	40.06±0.50	346	MQUc3-2	38.78±0.43	648	TZc3-14	45.63±0.26	950	ZKm5-2	44.21±1.02
45	HZc2-5	37.06±1.43	347	MQUc3-3	39.10±0.28	649	TZc3-15	48.68±0.34	951	ZKm5-3	44.19±0.83
46	HZc2-6	49.10±2.00	348	MQUc3-4	37.77±0.37	650	TZc3-16	43.75±0.62	952	ZKm5-4	40.43±0.84
47	HZc2-8	43.49±0.52	349	MQUc3-5	37.29±1.49	651	TZc3-17	46.87±1.05	953	ZKm5-5	38.59±0.42
48	HZc2-9	47.68±0.92	350	MQUc3-6	50.21±1.17	652	TZc3-18	45.72±0.18	954	ZKm5-6	40.85±1.06
49	HZc2-10	43.43±0.65	351	MQUc3-7	50.87±1.00	653	TZc3-19	49.94±0.74	955	ZKm5-7	40.73±0.85
50	HZc2-11	44.41±1.43	352	MQUc3-8	48.67±0.69	654	TZc3-20	52.51±0.66	956	ZKm5-8	37.53±1.01

51	HZc2-12	43.15±0.42	353	MQUc3-9	46.91±0.25	655	TZc3-21	49.78±0.63	957	ZKm5-9	41.48±0.78
52	HZc2-13	42.99±0.83	354	MQUc3-10	46.83±0.72	656	TZc3-22	48.03±0.78	958	ZKm6-1	44.70±2.03
53	HZc2-14	39.76±0.90	355	MQUc3-11	45.73±2.15	657	TZc3-23	50.91±0.18	959	ZKm6-2	45.24±0.73
54	HZc2-15	37.51±1.15	356	MQUc3-12	48.37±0.54	658	TZc3-24	51.53±0.07	960	ZKm7-1	43.72±4.57
55	HZc2-16	38.48±0.40	357	MQUc3-13	46.14±0.26	659	TZc3-25	49.13±0.24	961	ZKm7-2	48.32±1.49
56	HZc2-17	38.97±1.00	358	MQUc3-14	45.70±0.32	660	TZc5-1	47.45±1.15	962	ZKm7-3	48.08±2.76
57	HZc2-18	48.21±0.88	359	MQUc3-15	45.40±0.69	661	TZc5-2	47.69±0.05	963	ZKm7-4	43.22±2.61
58	HZc2-19	43.35±0.90	360	MQUc3-16	46.05±0.31	662	TZc5-3	48.75±0.52	964	ZKm7-5	45.85±0.78
59	HZc2-20	43.38±0.62	361	MQUc3-18	45.69±3.84	663	TZc5-4	47.48±0.10	965	ZKm7-6	47.60±2.23
60	HZc2-21	41.94±0.76	362	MQUc3-19	45.98±0.24	664	TZc5-5	47.55±0.85	966	ZKm7-7	44.17±1.37
61	HZc2-22	42.92±0.74	363	MQUc3-22	38.07±0.50	665	TZc5-7	47.48±0.74	967	ZKm7-8	44.19±2.70
62	HZc2-23	49.20±1.98	364	MQUc3-23	37.61±0.49	666	TZc5-8	48.05±1.28	968	ZKm7-9	44.33±2.69
63	HZc2-25	40.20±0.53	365	MQUc3-24	44.71±0.33	667	TZc5-10	47.86±0.92	969	ZKm7-10	48.48±2.52
64	HZc2-26	46.25±0.71	366	MQUc3-25	44.69±0.31	668	TZc5-11	47.78±0.84	970	ZKm7-11	45.25±0.70
65	HZc2-27	39.85±0.51	367	MQUc4-1	52.04±1.64	669	TZc5-12	49.29±1.61	971	ZKm7-12	43.16±1.98
66	HZc2-28	38.91±0.41	368	MQUc4-2	45.85±2.16	670	TZc5-13	51.19±1.43	972	ZKm7-13	44.48±1.63
67	HZc2-29	38.66±1.22	369	MQUc4-3	46.69±1.22	671	TZc5-15	46.20±0.40	973	ZKm7-14	47.81±3.18
68	HZc2-30	46.75±0.71	370	MQUc4-4	43.63±0.89	672	TZc5-16	47.97±1.90	974	ZKm7-15	40.44±3.55
69	HZc2-31	41.55±1.05	371	MQUc4-5	45.06±0.35	673	TZc5-18	48.50±0.87	975	ZKm7-16	41.46±2.18
70	HZc2-32	40.48±0.73	372	MQUc4-6	44.22±1.96	674	TZc5-19	47.51±1.22	976	ZKm7-17	29.92±7.59
71	HZc2-33	40.38±1.07	373	MQUc4-7	50.72±0.78	675	TZc5-20	47.27±1.75	977	ZKm7-18	50.92±1.92
72	HZc2-34	41.34±0.79	374	MQUc4-8	49.71±1.12	676	TZc5-21	47.61±0.70	978	ZKm7-19	43.95±4.90
73	HZc2-35	42.17±0.32	375	MQUc4-9	51.11±0.71	677	TZc5-22	47.02±0.29	979	ZKm8-1	49.54±0.73
74	HZc2-36	39.78±0.41	376	MQUc4-10	47.87±0.31	678	TZc5-23	46.42±0.46	980	ZKm8-2	49.06±0.85
75	HZc2-37	40.83±0.47	377	MQUc4-11	48.75±0.52	679	TZc5-24	45.02±0.21	981	ZKm8-3	39.40±1.24
76	HZc2-38	41.20±0.29	378	MQUc4-12	50.88±0.96	680	TZc6-1	49.49±2.03	982	ZKm8-4	58.35±1.80

77	HZc2-39	38.44±1.05	379	MQUc4-13	50.55±0.49	681	TZc6-2	48.30±0.46	983	ZKm8-5	51.21±1.46
78	HZc2-40	41.22±0.56	380	MQUc4-14	47.44±0.12	682	TZc6-3	49.13±0.32	984	ZKm8-6	52.66±0.61
79	HZc2-41	44.71±0.87	381	MQUc4-15	45.51±0.91	683	TZc6-4	48.23±0.89	985	ZKm8-7	51.77±0.43
80	HZc2-42	44.04±0.96	382	MQUc4-16	45.32±0.80	684	TZc6-5	48.33±0.26	986	ZKm9-1	42.22±0.92
81	HZc2-43	41.35±0.59	383	MQUc4-17	42.08±0.74	685	TZc6-6	47.88±0.30	987	ZKm9-2	45.76±2.03
82	HZc2-44	41.60±0.89	384	MQUc4-18	41.22±0.84	686	TZc7-2	50.62±2.46	988	ZKm9-3	46.04±0.07
83	HZc2-45	40.48±0.35	385	MQUc4-19	43.04±0.15	687	TZc7-4	48.54±0.76	989	ZKm9-4	42.48±0.86
84	HZc2-46	43.56±1.66	386	MQUc4-20	50.14±0.51	688	TZc7-5	49.14±2.01	990	ZKm9-5	40.17±2.00
85	HZc2-47	42.78±0.54	387	MQUc4-21	55.58±0.71	689	TZc7-6	52.31±2.83	991	ZKm9-6	45.76±0.85
86	HZc2-48	48.70±0.16	388	MQUc4-22	50.71±0.48	690	TZc7-7	52.39±0.78	992	ZKm9-7	37.38±0.55
87	HZc2-49	52.57±1.23	389	MQUc5-1	48.19±1.45	691	TZc7-8	51.51±0.84	993	ZKm9-8	38.02±1.68
88	HZc2-50	42.36±0.20	390	MQUc5-2	46.04±1.21	692	TZc7-9	50.24±1.05	994	ZKm9-9	38.15±0.96
89	HZc2-51	43.53±0.94	391	MQUc5-3	48.12±0.33	693	TZc8-1	53.30±0.60	995	MQm1-1	50.60±0.24
90	HZc2-52	42.64±0.48	392	MQUc5-4	42.29±1.28	694	TZc8-3	52.34±1.73	996	MQm1-2	49.42±0.79
91	HZc2-53	42.15±0.91	393	MQUc5-5	47.19±1.63	695	TZc8-4	51.40±0.80	997	MQm1-3	47.38±0.75
92	HZc2-54	41.52±0.57	394	MQUc5-6	44.68±0.73	696	TZc8-5	51.30±0.38	998	MQm1-4	50.69±0.38
93	XHEm1-1	51.59±1.48	395	MQUc5-7	48.36±1.24	697	TZc8-6	51.71±1.53	999	MQm1-5	44.55±1.54
94	XHEm1-2	48.38±2.91	396	MQUc5-8	46.26±1.21	698	TZc9-1	54.51±0.52	1000	MQm1-6	52.34±0.48
95	XHEm1-3	50.81±1.21	397	MQUc6-1	52.27±1.49	699	TZc9-2	53.36±0.44	1001	MQm1-7	49.84±0.73
96	XHEm1-4	54.20±0.40	398	MQUc6-2	49.59±0.87	700	TZc9-3	54.54±1.48	1002	MQm1-8	48.84±0.23
97	XHEm1-5	48.86±1.59	399	MQUc6-3	46.83±0.49	701	TZc9-4	51.64±1.30	1003	MQm2-1	44.93±1.22
98	XHEm1-6	50.86±0.75	400	MQUc6-4	47.24±0.35	702	TZc9-5	51.58±0.49	1004	MQm2-2	48.14±1.08
99	XHEm1-7	51.13±0.27	401	MQUc6-5	48.85±0.41	703	TZc9-6	52.50±0.21	1005	MQm2-3	48.96±0.50
100	XHEm1-8	51.17±1.16	402	MQUc6-6	47.96±2.82	704	TZc9-7	51.95±0.86	1006	MQm2-4	49.22±0.21
101	XHEm1-9	49.82±0.49	403	MQUc6-7	47.08±0.33	705	TZc9-8	51.29±0.25	1007	MQm2-5	48.21±0.52
102	XHEm1-10	51.11±1.31	404	MQUc6-8	47.10±0.50	706	TZc9-9	50.65±0.77	1008	MQm2-6	48.55±0.43

103	XHEm1-11	49.90±1.70	405	MQUc6-9	50.04±2.08	707	TZc9-10	51.52±0.34	1009	MQm2-7	51.48±2.98
104	XHEm1-12	44.41±0.76	406	MQUc6-10	48.76±0.74	708	TZc9-11	54.43±2.91	1010	MQm2-8	51.84±0.33
105	XHEm1-13	48.91±3.72	407	MQUc6-11	47.73±1.40	709	TZc9-12	52.35±1.93	1011	MQm3-1	54.94±0.99
106	XHEm1-14	50.05±3.34	408	MQUc6-12	47.77±0.55	710	TZc10-1	47.17±0.16	1012	MQm3-2	50.16±0.72
107	XHEm1-15	47.36±1.26	409	MQUc7-1	51.90±0.70	711	TZc10-2	42.68±0.22	1013	MQm3-3	50.33±2.01
108	XHEm1-16	47.90±1.17	410	MQUc7-2	55.16±1.60	712	TZc10-3	42.55±0.40	1014	MQm3-4	46.99±2.63
109	XHEm1-17	46.83±0.90	411	MQUc7-3	55.55±1.03	713	TZc10-6	46.12±0.71	1015	MQm3-5	53.30±1.48
110	XHEm1-18	47.13±0.28	412	MQUc7-4	52.36±1.34	714	TZc10-7	44.04±0.85	1016	MQm3-6	48.17±0.43
111	XHEm1-19	48.75±1.20	413	MQUc7-5	50.81±0.62	715	TZc10-8	44.16±0.33	1017	MQm3-7	50.94±1.16
112	XHEm1-20	47.81±0.31	414	MQUc7-6	52.20±1.87	716	TZc10-9	44.23±0.60	1018	MQm3-8	43.08±0.94
113	XHEc1-1	45.12±0.99	415	MQUc7-7	47.71±0.66	717	TZc10-10	46.41±0.61	1019	MQm3-9	51.38±0.30
114	XHEc1-2	44.30±0.35	416	MQUc7-8	42.96±0.39	718	SMy1-1	52.53±0.60	1020	MQm3-10	51.58±0.96
115	XHEc1-3	45.07±0.30	417	MQUc8-1	48.11±0.37	719	SMy1-2	52.24±1.75	1021	MQm3-11	44.73±1.46
116	XHEc1-4	45.83±0.19	418	MQUc8-2	47.46±0.71	720	SMy1-3	48.71±1.14	1022	MQm3-12	48.49±0.44
117	XHEc1-5	46.21±0.29	419	MQUc8-3	47.39±0.79	721	SMy1-4	49.98±1.59	1023	MQm4-1	50.48±0.47
118	XHEc1-6	46.69±0.52	420	MQUc8-4	45.70±0.43	722	SMm1-1	48.08±3.35	1024	MQm4-2	51.69±1.42
119	XHEc1-7	52.44±0.88	421	MQUc8-5	46.98±3.27	723	SMm1-2	47.83±1.04	1025	MQm4-3	52.17±1.69
120	XHEc1-8	51.34±0.80	422	MQUc8-6	47.25±0.44	724	SMm1-3	46.74±2.64	1026	MQm4-4	51.24±0.58
121	XHEc1-9	52.58±1.45	423	MQUc8-7	43.81±0.66	725	SMm1-4	48.64±0.27	1027	MQm4-5	56.73±2.95
122	XHEc1-10	57.10±2.55	424	MQUc8-8	43.12±0.64	726	SMm1-5	49.18±0.03	1028	MQm4-6	55.03±2.29
123	LQm1-1	47.07±0.96	425	MQUc8-9	43.24±0.75	727	SMm1-6	47.89±1.47	1029	MQm4-7	56.65±1.78
124	LQm1-2	51.58±1.78	426	MQUc8-10	45.92±0.18	728	SMm1-7	45.86±0.98	1030	MQm4-8	57.66±2.12
125	LQm1-3	44.53±0.48	427	MQUc9-1	50.12±0.58	729	SMm1-8	47.87±0.45	1031	MQm4-9	53.42±0.36
126	LQm1-4	48.92±0.54	428	MQUc9-2	50.03±0.81	730	SMm1-9	49.72±1.59	1032	MQm4-10	52.29±0.13
127	LQm1-5	49.46±0.65	429	MQUc9-3	47.88±0.17	731	SMm1-10	47.13±0.49	1033	MQm4-11	53.22±1.74
128	LQm1-6	48.94±1.39	430	MQUc9-4	48.13±0.23	732	SMm1-11	45.41±0.15	1034	GDm1-1	50.55±0.70

129	LQm1-7	47.85±0.40	431	MQUc9-5	48.72±0.25	733	SMm2-1	45.95±0.93	1035	GDm1-2	50.51±1.00
130	LQm1-8	47.64±1.73	432	MQUc9-6	47.89±0.61	734	SMm2-2	46.84±0.02	1036	GDm1-3	55.75±0.35
131	LQm1-9	47.57±0.10	433	MQUc9-7	52.34±0.81	735	SMm2-3	47.01±0.53	1037	GDm1-4	56.21±1.50
132	LQm2-1	54.76±3.10	434	MQUc9-8	54.37±0.79	736	SMm2-4	49.01±0.35	1038	GDm1-5	47.95±1.41
133	LQm2-2	54.18±0.84	435	MQUc9-9	55.60±1.63	737	SMm2-5	47.24±0.11	1039	GDm1-6	48.55±0.85
134	LQm2-3	55.27±2.32	436	MQUc9-10	53.56±0.48	738	SMm2-6	47.84±1.07	1040	GDm1-7	43.38±1.13
135	LQm2-4	58.49±1.64	437	MQUc9-11	53.43±0.48	739	SMm2-7	46.83±1.33	1041	GDm1-8	47.96±1.33
136	LQm2-5	53.79±2.32	438	MQUc9-12	52.94±0.19	740	SMm2-8	48.58±1.55	1042	GDm1-9	39.85±5.52
137	LQm2-6	55.78±1.23	439	MQUc9-13	53.08±0.76	741	SMm2-9	47.61±1.61	1043	GDm1-10	45.40±1.49
138	LQm2-7	55.20±0.45	440	MQUc9-14	53.04±0.73	742	SMm2-10	51.87±1.91	1044	GDm1-11	46.53±0.81
139	LQm2-8	56.71±1.44	441	MQUc9-15	52.55±1.04	743	SMm2-11	51.26±0.55	1045	GDm1-12	44.58±0.45
140	LQm2-9	51.66±0.13	442	MQUc9-16	52.74±0.39	744	SMm2-12	48.67±0.89	1046	GDm1-13	48.00±1.10
141	LQm2-10	51.46±0.36	443	MQUc9-17	52.33±0.70	745	SSm1-1	48.56±0.98	1047	GDm1-14	45.08±0.80
142	LQm2-11	51.89±0.79	444	MQUc9-18	52.19±0.62	746	SSm1-2	52.02±0.93	1048	GDm2-1	52.95±0.36
143	LQm2-12	52.49±0.66	445	MQUc9-19	52.39±0.66	747	SSm1-3	53.81±1.58	1049	GDm2-2	50.85±1.20
144	LQm2-13	50.28±0.75	446	MQUc9-20	51.64±0.22	748	SSm1-4	48.54±0.56	1050	GDm2-3	67.09±3.58
145	LQm2-14	50.22±0.69	447	MQUc9-21	52.25±0.40	749	SSm1-5	48.80±0.55	1051	GDm2-4	52.77±0.43
146	LQm2-15	51.65±0.45	448	MQUc9-22	51.82±0.30	750	SSm1-6	49.12±0.94	1052	GDm2-5	51.54±0.11
147	LQm2-16	51.52±1.20	449	MQUc9-23	51.70±0.77	751	SSm1-7	49.66±1.17	1053	GDm2-6	50.24±1.81
148	LQm2-17	50.56±0.55	450	MQUc9-24	50.84±0.46	752	SSm1-8	44.99±0.57	1054	GDm2-7	50.55±0.50
149	LQm2-18	50.38±0.35	451	MQUc9-25	51.68±0.36	753	SSm1-9	50.04±1.03	1055	GDm2-8	47.67±0.79
150	LQm2-19	50.60±0.44	452	MQUc9-26	52.10±0.89	754	SSm1-10	49.19±0.66	1056	GDm2-9	49.59±0.75
151	LQm2-20	51.24±0.94	453	MQUc9-27	53.85±1.58	755	SSm1-11	50.11±2.95	1057	GDm2-10	50.88±0.87
152	LQm2-21	45.96±1.87	454	MQUc9-28	52.96±0.56	756	SSm1-12	42.94±0.46	1058	GDm3-1	49.40±3.25
153	LQm2-22	50.29±0.49	455	MQUc9-29	53.51±0.73	757	SSm1-13	42.19±0.95	1059	GDm3-2	48.57±0.57
154	LQm2-23	50.56±0.14	456	MQUc9-30	52.48±1.21	758	SSm1-14	46.68±0.52	1060	GDm3-5	51.35±2.31

155	LQm2-24	52.16±1.06	457	MQUc9-31	51.56±0.73	759	SSm1-15	48.42±0.80	1061	GDy1-1	47.65±5.38
156	LQm2-25	51.64±1.83	458	MQUc9-32	51.26±0.20	760	SSm1-16	48.37±0.83	1062	GDy1-2	44.96±1.79
157	LQm2-27	51.84±0.25	459	MQUc9-33	52.66±0.27	761	SSm1-17	50.47±0.61	1063	GDc1-1	40.43±1.25
158	LQm2-28	51.54±1.09	460	MQUc9-34	52.41±0.42	762	SSm1-18	50.58±0.86	1064	GDc1-2	37.31±2.28
159	LQm2-29	50.37±1.74	461	MQUc9-35	52.69±0.41	763	SSm1-19	51.14±1.04	1065	GDc1-3	39.58±2.92
160	LQm2-30	49.51±1.51	462	MQUc9-36	49.01±0.46	764	XHm1-1	42.01±1.26	1066	GDc1-4	38.48±0.84
161	LQm2-31	51.89±3.31	463	MQUc9-37	45.62±0.77	765	XHm1-2	42.77±0.74	1067	GDc1-5	38.41±1.27
162	LQm2-32	51.69±5.12	464	MQUc9-38	47.65±0.44	766	XHm1-3	50.89±2.31	1068	GDc1-6	39.64±1.51
163	LQm2-33	47.42±0.84	465	MQUc9-39	49.52±0.23	767	XHm1-4	50.04±2.42	1069	GDc1-7	37.13±0.90
164	LQc1-1	50.40±0.41	466	MQUc9-40	49.33±0.11	768	XHm1-5	54.73±3.23	1070	GDc1-8	40.59±0.89
165	LQc1-2	49.43±0.55	467	MQUc10-1	50.36±0.64	769	XHm1-6	57.52±2.77	1071	GDc1-9	36.97±0.39
166	LQc2-1	46.95±0.41	468	MQUc10-2	51.31±0.52	770	XHm1-7	48.59±0.93	1072	GDc1-10	35.47±2.54
167	LQc2-2	47.44±1.21	469	MQUc10-3	49.61±0.91	771	XHm1-8	50.13±2.30	1073	GDc1-11	36.56±1.06
168	LQc2-3	49.30±0.52	470	MQUc10-4	50.49±1.92	772	XHm2-1	39.37±0.66	1074	GDc1-12	37.18±1.85
169	LQc2-4	47.20±0.61	471	MQUc10-5	54.08±4.64	773	XHm2-2	41.87±0.82	1075	GDc1-13	39.58±2.76
170	LQc2-5	48.40±0.73	472	MQUc10-6	51.04±0.57	774	XHm2-3	41.70±1.91	1076	GDc1-14	37.97±1.24
171	LQc2-6	48.47±1.04	473	JZc1-1	47.52±0.47	775	XHm2-4	43.19±2.43	1077	GDc1-15	38.96±1.46
172	LQc2-7	43.89±1.60	474	JZc1-2	45.02±0.78	776	XHm2-5	41.41±1.79	1078	GDc1-16	37.16±0.91
173	LQc2-8	47.35±0.21	475	JZc1-3	46.47±0.26	777	XHm2-6	39.95±2.54	1079	GDc1-17	35.60±0.57
174	LQc2-9	47.95±0.21	476	JZc1-4	46.52±0.99	778	XHm2-7	42.24±1.74	1080	GDc1-18	59.00±1.54
175	LQc2-10	46.89±0.71	477	JZc1-5	46.60±0.39	779	XHm2-8	40.20±0.89	1081	TDc1-1	43.88±0.79
176	LQc2-11	46.23±0.61	478	JZc1-6	60.71±0.64	780	XHm2-9	41.36±5.02	1082	TDc2-1	54.06±2.48
177	LQc2-12	50.31±1.46	479	JZc1-7	63.49±0.52	781	XHm3-1	51.86±0.63	1083	TDc2-2	51.61±1.11
178	LQc2-13	47.85±1.83	480	JZc1-8	64.16±1.28	782	XHm3-2	52.86±1.30	1084	TDc2-3	51.53±0.33
179	LQc2-14	48.22±0.99	481	JZc1-9	65.00±1.31	783	XHm3-3	52.85±0.75	1085	TDc2-4	53.01±0.34
180	MQUy2-1	47.36±1.02	482	JZc1-10	59.54±0.25	784	XHm3-4	52.31±0.43	1086	TDc2-5	50.71±2.71

181	MQUy2-2	42.36±0.35	483	JZc1-11	58.52±1.57	785	XHm4-1	53.57±0.37	1087	TDc2-6	47.20±1.00
182	MQUy2-3	44.06±0.98	484	JZc1-12	60.94±1.66	786	XHm4-2	54.26±0.54	1088	TDc2-7	49.25±0.41
183	MQUy2-4	46.06±2.20	485	JZc1-13	60.83±0.53	787	XHm4-3	53.41±0.47	1089	TDc2-8	52.37±0.42
184	MQUy2-5	43.89±1.14	486	JZc1-14	59.17±0.96	788	XHm4-4	52.82±0.32	1090	TDc2-9	52.48±1.28
185	MQUy2-6	42.51±0.44	487	JZc1-15	57.29±0.37	789	XHm4-5	51.01±4.97	1091	TDc2-10	43.43±1.81
186	MQUy2-7	43.72±0.42	488	JZc1-16	57.97±0.27	790	XHm4-6	52.37±0.50	1092	TDc2-11	43.41±1.19
187	MQUy2-8	47.78±0.42	489	JZc1-17	59.38±0.26	791	XHm4-7	49.67±0.55	1093	TDc2-12	43.54±1.58
188	MQUy2-9	42.74±1.00	490	JZc1-18	54.27±0.56	792	XHm4-8	49.75±0.69	1094	TDc2-13	45.38±2.66
189	MQUy2-10	43.10±1.10	491	ZNy1-1	45.55±0.70	793	XHm4-9	51.15±1.49	1095	TDc2-14	38.72±1.38
190	MQUy2-11	43.15±0.78	492	ZNy1-2	44.55±1.05	794	XHm4-10	50.06±0.53	1096	TDc2-15	46.28±2.56
191	MQUy2-12	43.16±0.29	493	ZNy1-3	46.77±2.64	795	XHm4-11	49.25±2.35	1097	QL01	53.02±0.55
192	MQUy2-13	42.99±1.15	494	ZNy1-4	46.06±1.50	796	XHm5-1	51.27±1.05	1098	QLm1-2	44.98±0.06
193	MQUy2-14	43.24±0.66	495	ZNy1-5	46.04±0.69	797	XHm5-2	48.87±1.41	1099	QLm1-3	46.34±2.62
194	MQUy2-15	43.30±0.95	496	ZNc1-1	51.96±0.33	798	XHm5-3	49.75±2.07	1100	QLm1-4	44.66±0.56
195	MQUy2-16	44.80±1.30	497	ZNc1-2	50.85±2.86	799	XHm5-4	46.47±0.73	1101	QLm1-5	45.29±0.76
196	MQUy3-1	42.02±1.92	498	ZNc1-3	53.87±0.99	800	XHm5-5	51.04±2.93	1102	QLm1-6	45.14±1.05
197	MQUy3-2	47.34±2.56	499	ZNc1-4	54.20±0.97	801	XHm5-6	48.02±0.41	1103	QLm1-7	45.38±1.72
198	MQUy3-3	48.91±0.90	500	ZNc1-5	52.73±1.63	802	XHm5-7	51.18±1.03	1104	QLm1-8	42.08±0.56
199	MQUy3-4	49.87±0.31	501	ZNc1-6	53.36±0.51	803	XHm5-8	56.00±1.52	1105	QLm1-9	43.55±0.35
200	MQUy3-5	51.05±1.86	502	ZNc1-7	53.54±0.63	804	XHm5-9	55.51±1.34	1106	QLm1-10	51.61±6.09
201	MQUy3-6	44.38±1.99	503	ZNc1-8	43.80±3.88	805	XHm5-10	55.83±0.56	1107	QLm1-11	45.82±0.93
202	MQUy3-7	50.52±0.95	504	ZNc1-9	49.68±1.98	806	XHm5-11	52.60±1.02	1108	QLm1-12	47.10±0.36
203	MQUy3-7	49.69±0.57	505	ZNc1-10	49.36±0.58	807	XHm5-12	55.78±0.70	1109	QLm1-13	46.80±0.24
204	MQUy3-8	52.22±0.57	506	ZNc1-11	49.56±0.64	808	XHm5-13	55.53±1.29	1110	QLm1-14	43.60±0.58
205	MQUy3-9	53.14±0.53	507	ZNc1-12	50.45±1.18	809	XHm5-14	49.96±0.90	1111	QLm1-15	44.84±0.25
206	MQUy3-10	51.24±0.69	508	ZNc1-13	45.85±0.62	810	XHm5-15	52.15±0.23	1112	QLm1-16	44.01±0.13

207	MQUy3-11	47.86±0.63	509	ZNc1-14	46.65±0.16	811	XHm5-16	49.94±1.38	1113	QLm1-17	46.23±0.40
208	MQUy3-12	47.80±1.11	510	TZy1-1	47.21±1.66	812	XHm5-17	55.16±0.34	1114	QLm1-18	47.12±2.87
209	MQUy3-13	51.50±3.06	511	TZy1-2	46.87±0.40	813	XHm5-18	54.87±0.53	1115	QLm1-19	47.42±0.30
210	MQUy3-14	45.54±0.85	512	TZy1-3	46.65±0.81	814	XHm5-19	48.59±0.44	1116	QLm1-20	41.50±0.67
211	MQUy3-15	46.51±2.72	513	TZy1-4	44.28±0.41	815	XHm6-1	48.35±0.85	1117	QLm1-21	43.41±1.21
212	MQUy3-16	48.37±0.43	514	TZy1-5	46.30±1.18	816	XHm6-2	53.63±0.35	1118	QLm1-22	48.04±0.28
213	MQUy4-1	50.57±0.28	515	TZy1-6	47.03±0.88	817	XHm6-3	45.68±1.32	1119	QLm2-1	35.03±3.11
214	MQUy4-2	49.80±0.80	516	TZy1-7	46.32±0.65	818	XHm6-4	45.87±0.07	1120	QLm2-2	45.05±0.35
215	MQUy4-3	49.58±0.91	517	TZy1-8	47.93±1.90	819	XHm6-5	47.85±0.45	1121	QLm2-3	43.84±0.16
216	MQUy4-4	50.18±1.07	518	TZy1-9	42.79±0.66	820	XHm6-6	50.59±2.07	1122	QLm2-4	45.24±1.09
217	MQUy4-5	50.06±0.76	519	TZy2-1	50.37±0.60	821	XHm6-7	45.83±2.36	1123	QLm2-5	45.38±1.35
218	MQUy4-6	49.44±1.89	520	TZy2-2	48.14±0.69	822	XHm6-8	45.48±0.15	1124	QLm2-6	48.52±0.35
219	MQUy4-7	45.57±1.30	521	TZy2-3	49.27±0.51	823	XHm7-1	52.51±3.62	1125	QLm2-7	48.21±0.55
220	MQUy4-8	43.73±0.49	522	TZy2-4	47.38±1.40	824	XHm7-2	64.14±3.06	1126	QLm2-8	48.49±0.71
221	MQUy4-9	41.13±0.83	523	TZy2-5	50.32±1.46	825	XHm7-3	51.38±1.32	1127	QLm2-9	49.51±0.57
222	MQUy4-10	48.12±1.19	524	TZy2-6	49.76±1.39	826	XHm7-4	50.51±0.29	1128	QLm2-10	50.44±1.21
223	MQUy4-11	45.46±2.84	525	TZy2-7	51.19±0.81	827	XHm7-5	50.32±2.23	1129	QLm2-11	49.83±0.36
224	MQUy4-12	47.22±1.10	526	TZy2-8	49.46±0.74	828	XHm7-6	48.74±1.60	1130	QLm2-12	46.74±0.75
225	MQUy4-13	47.61±0.98	527	TZy2-9	49.34±0.31	829	XHm7-7	47.86±0.60	1131	QLm2-13	49.28±0.29
226	MQUy4-14	45.32±0.77	528	TZy3-1	49.20±3.77	830	XHm7-8	49.43±1.44	1132	QLm2-14	43.62±0.61
227	MQUy4-15	46.37±1.38	529	TZy3-2	49.04±0.35	831	XHm7-9	47.48±1.37	1133	QLm2-15	44.06±1.56
228	MQUy4-16	46.23±2.58	530	TZy3-3	52.72±1.12	832	XHm7-10	51.33±2.24	1134	QLm2-16	40.84±1.10
229	MQUy4-17	47.08±1.74	531	TZy3-4	52.27±1.56	833	XHm7-11	48.73±0.48	1135	QLm2-17	53.10±0.83
230	MQUy4-18	46.48±0.76	532	TZy4-1	47.93±2.14	834	XHm7-12	50.41±1.96	1136	QLm2-18	48.05±0.19
231	MQUy4-19	44.85±0.43	533	TZy4-2	48.96±0.27	835	XHm7-13	48.74±1.15	1137	QLm2-19	48.49±1.50
232	MQUy4-20	47.24±0.87	534	TZy4-3	47.36±0.97	836	XHm7-14	51.25±2.39	1138	QLm2-20	51.98±0.94

233	MQUy4-21	44.69±1.53	535	TZy4-4	48.53±0.88	837	XHm8-1	49.85±0.59	1139	QLm3-1	35.00±1.60
234	MQUy4-22	46.27±0.55	536	TZy4-5	48.85±1.70	838	XHm8-2	51.26±0.15	1140	QLm3-2	51.06±3.82
235	MQUy4-23	42.87±1.06	537	TZy4-6	46.85±0.77	839	XHm8-3	52.06±1.98	1141	QLm3-3	45.99±1.07
236	MQUy4-24	45.10±0.84	538	TZy6-1	50.64±0.56	840	XHm8-4	51.29±0.55	1142	QLm3-4	44.13±0.24
237	MQUy4-25	44.02±1.13	539	TZy6-2	50.66±0.92	841	XHm8-5	52.12±1.45	1143	QLm3-5	46.59±1.37
238	MQUy4-26	50.83±3.30	540	TZy6-3	52.51±0.72	842	XHm8-6	50.59±0.73	1144	QLm3-6	51.94±5.78
239	MQUy4-27	49.20±0.63	541	TZy6-4	52.31±0.95	843	XHm8-7	51.18±0.36	1145	QLm3-7	39.52±1.81
240	MQUy4-28	47.28±0.67	542	TZy6-5	53.87±1.52	844	XHm8-8	51.12±0.24	1146	QLm3-8	46.87±0.63
241	MQUy4-29	47.81±0.85	543	TZy6-6	49.93±0.49	845	XHc1-1	45.15±0.41	1147	QLm3-9	37.62±0.30
242	MQUy4-30	46.39±0.42	544	TZy6-7	52.05±1.48	846	XHc2-1	42.87±1.84	1148	QLm3-10	36.05±5.02
243	MQUy4-31	48.27±0.31	545	TZy6-8	51.82±1.74	847	XHc2-2	44.44±0.90	1149	QLm3-11	30.12±3.52
244	MQUy4-32	47.80±1.02	546	TZm1-1	41.91±0.39	848	XHc2-3	45.74±0.57	1150	QLm3-12	17.37±0.34
245	MQUy4-33	46.95±1.03	547	TZm1-2	44.31±1.15	849	XHc2-4	47.89±1.03	1151	QLm3-13	40.68±2.08
246	MQUy4-34	46.63±0.61	548	TZm1-3	48.97±1.96	850	XHc2-5	45.20±0.85	1152	QLm3-14	40.23±3.90
247	MQUy4-35	45.46±0.93	549	TZm1-4	41.85±1.83	851	XHc2-6	38.68±3.41	1153	QLm3-15	31.43±1.30
248	MQUy4-36	46.63±0.55	550	TZm1-5	44.15±0.40	852	XHc2-7	38.79±3.08	1154	QLm3-16	28.64±0.33
249	MQUy4-37	45.00±2.46	551	TZm1-6	43.88±0.99	853	HNm1-1	52.51±0.34	1155	QLm3-17	36.29±1.86
250	MQUy4-38	45.24±1.28	552	TZm1-7	42.94±0.73	854	HNm1-2	50.72±0.58	1156	QLm3-18	44.45±1.74
251	MQUy4-39	46.16±0.90	553	TZm1-8	40.69±0.76	855	HNm1-3	51.95±0.59	1157	QLm3-19	28.99±2.95
252	MQUy5-1	51.22±0.68	554	TZm1-9	42.67±1.61	856	HNm1-4	50.14±0.62	1158	QLm3-20	27.32±0.40
253	MQUy5-2	51.31±2.20	555	TZm1-10	39.65±0.64	857	HNm1-5	50.74±0.63	1159	QLm4-1	39.41±0.92
254	MQUy7-1	43.37±0.44	556	TZm1-11	46.36±0.58	858	HNm1-6	50.79±0.94	1160	QLm4-2	45.50±0.17
255	MQUy7-2	43.17±1.02	557	TZm1-12	46.07±1.14	859	HNm1-7	42.13±2.30	1161	QLm4-3	48.36±0.14
256	MQUy7-3	48.21±0.65	558	TZm1-13	43.12±1.34	860	HNm1-8	45.63±0.60	1162	QLm4-4	48.86±3.63
257	MQUy7-4	45.47±1.48	559	TZm1-14	47.77±1.39	861	HNm2-1	45.65±1.65	1163	QLm4-5	48.76±2.95
258	MQUy7-5	44.12±0.49	560	TZm2-1	49.18±1.99	862	HNm2-2	45.77±0.90	1164	QLm4-6	48.09±3.04

259	MQUy7-6	44.75±1.49	561	TZm2-2	50.84±1.58	863	HNm2-3	50.84±1.75	1165	QLm4-7	50.46±1.40
260	MQUy7-7	49.49±2.14	562	TZm2-3	50.52±1.02	864	HNm2-4	50.28±0.28	1166	QLm4-8	46.64±1.63
261	MQUy7-8	46.07±1.35	563	TZm2-4	50.58±0.91	865	HNm2-5	50.00±1.05	1167	QLm4-9	44.39±0.34
262	MQUy9-1	44.69±0.25	564	TZm2-5	51.37±0.69	866	HNm2-6	48.83±0.53	1168	QLm4-10	47.68±0.77
263	MQUy9-2	45.26±0.91	565	TZm2-6	46.60±1.31	867	HNm2-7	49.53±0.54	1169	QLm4-11	44.58±0.79
264	MQUy9-3	52.90±3.89	566	TZm2-7	48.47±0.35	868	HNm2-8	50.21±0.94	1170	QLm4-12	48.46±0.55
265	MQUy9-4	42.49±1.71	567	TZm2-8	49.50±0.57	869	HNm3-1	47.05±1.32	1171	QLm4-13	51.22±2.30
266	MQUy9-5	45.78±0.97	568	TZm2-9	51.66±3.57	870	HNm3-2	47.26±0.77	1172	QLm4-14	44.44±1.17
267	MQUy9-6	45.36±1.91	569	TZm3-1	46.58±2.68	871	HNm3-3	49.54±0.82	1173	QLm4-15	53.49±8.40
268	MQUy9-7	43.40±1.45	570	TZm3-2	44.01±1.13	872	HNm3-4	49.22±0.90	1174	QLm4-16	42.63±1.06
269	MQUy10-1	44.24±0.43	571	TZm3-3	46.11±1.71	873	HNm3-6	47.42±0.23	1175	QLm4-17	46.24±0.48
270	MQUy10-2	49.48±0.11	572	TZm3-4	51.65±3.19	874	HNm3-7	48.02±0.43	1176	QLm4-18	48.09±4.51
271	MQUy10-3	44.45±1.56	573	TZm3-5	46.40±1.37	875	HNm3-8	48.55±0.70	1177	QLm4-19	42.64±0.95
272	MQUy10-4	49.70±0.32	574	TZm4-1	51.06±0.30	876	HNm4-1	54.95±2.72	1178	QLm4-20	45.00±0.81
273	MQUy10-5	48.99±0.53	575	TZm4-2	52.35±0.21	877	HNm4-4	52.29±1.77	1179	QLm5-1	50.01±1.25
274	MQUy10-7	48.64±0.42	576	TZm4-3	51.03±2.27	878	HNm4-3	43.44±0.77	1180	QLm5-2	52.60±0.97
275	MQUy10-8	49.73±0.70	577	TZm4-4	49.79±0.47	879	HNm5-1	36.61±0.43	1181	QLm5-3	46.44±0.93
276	MQUy10-9	50.38±0.72	578	TZm4-5	46.39±0.44	880	HNm6-1	50.91±2.19	1182	QLm5-4	44.13±1.33
277	MQUy10-10	50.18±0.82	579	TZm4-6	48.98±0.40	881	HNm6-2	49.71±1.39	1183	QLm5-5	45.99±1.97
278	MQUy10-11	50.13±1.16	580	TZm4-7	48.78±0.16	882	HNm6-3	52.07±0.68	1184	QLm5-6	51.76±0.43
279	MQUy10-12	50.08±0.83	581	TZm4-8	48.75±0.21	883	HNm6-4	50.00±0.89	1185	QLm5-7	50.37±0.46
280	MQUm1-1	52.12±0.45	582	TZm4-9	50.40±0.78	884	HNm6-5	48.16±0.29	1186	QLm5-8	50.19±0.18
281	MQUm1-2	51.60±0.40	583	TZm4-10	46.98±0.15	885	HNm6-6	50.43±0.00	1187	QLm5-9	50.03±1.84
282	MQUm1-3	52.12±0.84	584	TZc1-1	51.52±3.47	886	HNy1-1	45.34±0.98	1188	QLm5-10	46.95±4.27
283	MQUm1-4	52.52±1.37	585	TZc1-2	57.89±1.39	887	HNy1-2	46.07±0.80	1189	QLm5-11	39.77±2.53
284	MQUm1-5	49.22±0.82	586	TZc1-3	56.86±0.41	888	HNy1-3	44.29±0.46	1190	QLm5-12	42.77±2.60

285	MQUm1-6	49.12±0.57	587	TZc1-4	56.76±1.02	889	HNy1-4	44.54±1.08	1191	QLm5-13	38.11±0.82
286	MQUm1-7	48.07±0.94	588	TZc1-5	57.14±1.20	890	HNy2-1	40.73±0.53	1192	QLm5-14	39.01±1.18
287	MQUm1-8	47.85±0.36	589	TZc1-6	56.53±4.45	891	HNy2-2	42.70±0.32	1193	SZ-1	44.80±0.90
288	MQUm1-9	46.16±0.76	590	TZc1-7	56.02±0.41	892	HNy2-3	42.41±1.05	1194	SZ-2	42.66±0.25
289	MQUm1-10	49.97±0.25	591	TZc1-8	55.39±0.39	893	HNy4-1	45.90±0.44	1195	SZ-3	49.48±4.06
290	MQUm2-1	56.00±0.26	592	TZc1-9	54.78±1.00	894	HNy4-2	50.24±2.27	1196	SZ-4	49.14±0.93
291	MQUm2-2	49.92±0.16	593	TZc1-10	54.49±1.22	895	HNy4-3	49.27±1.70	1197	SZ-5	46.82±0.50
292	MQUm2-3	49.93±1.22	594	TZc1-11	52.46±0.04	896	HNy4-4	57.17±3.73	1198	SZ-6	47.93±0.78
293	MQUm2-4	46.84±1.75	595	TZc1-12	54.46±0.20	897	HNy4-5	63.84±3.28	1199	SZ-7	47.49±0.65
294	MQUm2-5	50.35±1.89	596	TZc1-13	55.22±1.51	898	HNy4-6	50.79±0.13	1200	SZ-8	41.49±0.35
295	MQUm2-6	51.83±0.92	597	TZc1-14	49.41±1.28	899	HNy4-7	50.65±0.10	1201	SZ-9	42.53±0.51
296	MQUm2-7	50.03±1.74	598	TZc1-15	52.54±0.62	900	HNy5-1	51.13±0.23	1202	SZ-10	41.21±0.88
297	MQUm2-8	49.78±3.76	599	TZc1-16	53.08±2.10	901	HNy5-2	52.01±0.43	1203	SZ-11	45.16±6.58
298	MQUm2-9	48.78±0.34	600	TZc2-1	52.55±0.21	902	HNy5-3	52.48±0.52	1204	SZ-12	41.83±0.96
299	MQUm2-10	50.69±1.91	601	TZc2-2	52.13±0.69	903	HNy5-4	46.13±1.14	1205	SZ-13	40.73±2.21
300	MQUm2-11	50.33±3.24	602	TZc2-4	51.62±0.58	904	HNy5-5	52.84±0.99			
301	MQUm2-12	47.88±0.93	603	TZc2-5	56.97±0.27	905	HNy5-6	51.87±0.29			
302	MQUm2-13	48.13±0.42	604	TZc2-6	56.62±0.42	906	HNy5-7	52.38±0.97			

Values are mean ± SD (n = 3).

Table S2. Growth of lactic acid bacteria at different concentrations of H_2O_2.

	Strain	Concentration of H_2O_2			
		**0 mmol/L**	**1 mmol/L**	**2 mmol/L**	**3 mmol/L**
1	MQUy9-3	1.261±0.03	1.255±0.00	1.248±0.02	1.126±0.03
2	QL01	1.403±0.03	1.365±0.02	1.372±0.01	1.356±0.00
3	TZc2-17	1.395±0.03	1.337±0.01	1.311±0.02	1.288±0.04
4	TZc2-18	1.247±0.04	1.162±0.03	1.216±0.05	1.203±0.03
5	TZc2-19	1.207±0.06	1.232±0.07	1.266±0.09	1.130±0.06
6	TZc2-20	1.233±0.00	1.259±0.01	1.207±0.04	1.250±0.02
7	TZc2-21	1.377±0.02	1.367±0.03	1.348±0.02	1.318±0.03
8	TZc2-22	1.343±0.01	1.369±0.01	1.378±0.02	1.334±0.02
9	TZc2-23	1.341±0.02	1.314±0.02	1.324±0.02	1.254±0.01
10	TZc2-24	1.257±0.02	1.261±0.02	1.247±0.02	1.256±0.05
11	TZc2-26	1.312±0.05	1.231±0.03	1.187±0.03	1.189±0.02
12	ZNc1-5	1.064±0.02	1.050±0.01	1.070±0.00	1.104±0.03
13	HZc1-19	0.485±0.00	0.472±0.00	0.473±0.01	-
14	JZc1-10	1.122±0.01	1.061±0.03	-	-
15	JZc1-11	1.112±0.01	1.093±0.02	-	-
16	JZc1-12	1.086±0.03	1.053±0.01	-	-
17	JZc1-13	1.171±0.01	1.150±0.02	-	-
18	JZc1-14	1.129±0.02	1.074±0.02	-	-
19	JZc1-15	1.129±0.03	1.101±0.02	-	-
20	JZc1-17	1.112±0.01	1.082±0.01	-	-
21	JZc1-18	1.133±0.03	1.089±0.02	-	-
22	JZc1-6	1.134±0.01	1.087±0.02	-	-
23	HZy1-3	0.324±0.01	0.318±0.00	-	-
24	MQUc7-2	0.260±0.00	0.265±0.01	0.258±0.06	-
25	MQUc7-3	0.302±0.00	0.289±0.00	0.253±0.00	-
26	GDm1-3	0.444±0.01	0.412±0.00	0.406±0.00	-
27	GDm1-4	0.469±0.00	0.459±0.00	0.362±0.01	-
28	HNm4-1	0.433±0.00	0.431±0.01	0.412±0.01	-
29	HNy6-1	0.359±0.00	0.377±0.00	0.304±0.01	-
30	HNy6-3	0.350±0.01	0.315±0.02	0.324±0.01	-
31	MQUc4-21	0.472±0.00	0.476±0.00	0.450±0.01	-
32	MQUc10-5	0.475±0.01	0.478±0.00	0.426±0.00	-
33	MQm3-1	0.422±0.01	0.417±0.01	0.393±0.00	-
34	MQm3-5	0.399±0.00	0.390±0.00	0.369±0.01	-
35	MQm4-11	0.399±0.00	0.378±0.00	0.371±0.00	-
36	MQm4-5	0.472±0.00	0.462±0.01	0.424±0.03	-
37	MQm4-6	0.382±0.01	0.386±0.01	0.343±0.01	-
38	MQm4-8	0.391±0.01	0.402±0.00	0.354±0.00	-
39	MQm4-9	0.440±0.01	0.425±0.00	0.400±0.00	-
40	QLm2-17	0.479±0.01	0.467±0.01	0.437±0.01	-
41	SSm1-3	0.322±0.01	0.319±0.01	0.267±0.00	-

42	XHm5-10	0.392±0.01	0.386±0.01	0.338±0.00	-
43	XHm5-12	0.395±0.00	0.396±0.01	-	-
44	XHm5-13	0.360±0.01	0.354±0.01	0.408±0.01	-
45	XHm5-17	0.388±0.01	0.389±0.01	0.385±0.01	-
46	XHm5-18	0.429±0.00	0.425±0.01	0.467±0.01	-
47	XHm5-9	0.401±0.00	0.405±0.01	0.423±0.01	-
48	XHm6-2	0.401±0.01	0.385±0.01	0.389±0.00	-
49	ZKm8-4	0.380±0.00	0.379±0.00	0.352±0.00	-
50	GDm2-1	0.397±0.01	0.399±0.00	0.356±0.01	0.171±0.00
51	HNy4-4	0.316±0.01	0.311±0.01	0.299±0.01	-
52	HNy4-5	0.343±0.01	0.342±0.00	0.315±0.00	-
53	HNy5-5	0.381±0.01	0.378±0.01	0.342±0.01	0.338±0.01
54	JZc1-16	0.437±0.01	0.431±0.01	0.412±0.00	-
55	JZc1-9	0.372±0.00	0.339±0.01	0.313±0.00	-
56	LQm2-5	0.395±0.01	0.379±0.00	0.340±0.01	0.215±0.01
57	LQm2-6	0.430±0.01	0.434±0.02	0.410±0.01	0.203±0.02
58	LQm2-7	0.358±0.01	0.380±0.02	0.345±0.01	0.242±0.01
59	LQm2-8	0.325±0.01	0.315±0.00	0.282±0.00	0.112±0.00
60	MQUc9-12	0.451±0.00	0.457±0.01	0.456±0.01	-
61	MQUc9-13	0.425±0.01	0.425±0.01	0.425±0.02	-
62	MQUc9-14	0.43±0.01	0.409±0.04	0.425±0.01	-
63	MQUc9-15	0.436±0.03	0.408±0.02	0.422±0.01	-
64	MQUc9-27	0.435±0.01	0.453±0.02	0.476±0.04	-
65	MQUc9-28	0.442±0.01	0.449±0.02	0.448±0.01	-
66	MQUc9-29	0.436±0.01	0.428±0.01	0.425±0.01	-
67	MQUc9-8	0.489±0.01	0.476±0.01	0.504±0.01	-
68	MQUc9-9	0.460±0.00	0.458±0.00	0.434±0.01	-
69	TZc1-12	0.488±0.01	0.484±0.00	0.509±0.01	-
70	TZc1-13	0.484±0.00	0.482±0.00	0.513±0.00	-
71	TZc1-8	0.546±0.01	0.522±0.01	0.498±0.01	-
72	XHm1-5	0.400±0.00	0.394±0.00	0.385±0.01	-
73	XHm1-6	0.399±0.01	0.395±0.01	0.393±0.01	0.354±0.01
74	XHm3-2	0.400±0.01	0.386±0.01	0.368±0.01	-
75	XHm4-2	0.444±0.02	0.415±0.01	0.390±0.01	0.396±0.01
76	XHm7-2	0.363±0.01	0.356±0.00	0.332±0.00	-
77	XHEm1-4	0.406±0.01	0.415±0.01	0.382±0.00	-
78	HZc1-1	0.400±0.02	0.416±0.01	0.434±0.01	0.474±0.01
79	HZc1-2	0.439±0.00	0.451±0.01	0.49±0.00	0.512±0.01
80	LQm2-1	0.458±0.00	0.489±0.00	0.494±0.00	0.525±0.01
81	LQm2-2	0.476±0.00	0.488±0.01	0.515±0.00	0.540±0.00
82	LQm2-3	0.428±0.00	0.422±0.02	0.44±0.04	0.477±0.01
83	LQm2-4	0.406±0.00	0.418±0.01	0.434±0.00	0.476±0.00
84	MQUc9-10	0.459±0.01	0.464±0.00	0.476±0.01	0.532±0.00
85	MQUc9-11	0.494±0.00	0.515±0.00	0.542±0.00	0.552±0.01

86	TDc2-1	0.501±0.01	0.510±0.01	0.504±0.01	0.535±0.01
87	TZc1-2	0.386±0.00	0.413±0.01	0.426±0.00	0.476±0.01
88	TZc1-5	0.399±0.01	0.406±0.01	0.395±0.02	0.450±0.02
89	TZc1-7	0.419±0.00	0.448±0.00	0.481±0.01	0.483±0.01
90	TZc1-9	0.410±0.00	0.449±0.00	0.456±0.01	0.499±0.01
91	TZc2-15	0.48±0.02	0.389±0.08	0.481±0.01	0.501±0.02
92	TZc2-16	0.485±0.04	0.484±0.07	0.468±0.00	0.493±0.02
93	TZc2-5	0.454±0.02	0.434±0.03	0.474±0.00	0.503±0.00
94	XHEc1-10	0.434±0.01	0.449±0.01	0.464±0.01	0.504±0.01
95	XHm5-8	0.474±0.01	0.484±0.00	0.498±0.01	0.533±0.01
96	ZNc1-3	0.481±0.01	0.506±0.01	0.521±0.00	0.527±0.01
97	ZNc1-4	0.470±0.01	0.490±0.01	0.512±0.01	0.514±0.00
98	ZNc1-6	0.497±0.02	0.494±0.01	0.542±0.00	0.548±0.02
99	ZNc1-7	0.495±0.00	0.522±0.00	0.560±0.00	0.543±0.00
100	GDm2-3	0.830±0.02	0.887±0.01	0.819±0.00	-
101	XHm4-4	0.935±0.01	0.968±0.01	0.931±0.02	0.880±0.02
102	GDc1-18	0.527±0.01	0.547±0.01	0.122±0.00	-
103	MQUy3-10	0.278±0.00	0.27±0.00	0.173±0.00	-
104	MQm4-7	0.249±0.01	0.232±0.00	0.168±0.00	-
105	TZy6-5	0.269±0.00	0.262±0.00	0.183±0.00	-
106	GDm2-4	0.402±0.01	0.386±0.01	0.386±0.01	-
107	HZy3-1	0.456±0.01	0.442±0.01	0.452±0.01	0.115±0.01
108	TDc2-4	0.425±0.02	0.415±0.01	0.405±0.01	0.332±0.00
109	TZc1-3	0.406±0.01	0.402±0.00	0.407±0.00	0.091±0.00
110	TZc1-4	0.374±0.06	0.449±0.04	0.400±0.03	-
111	XHm3-3	0.422±0.01	0.424±0.00	0.401±0.01	0.092±0.01
112	XHm4-1	0.455±0.01	0.448±0.00	0.412±0.01	0.155±0.00
113	XHm4-3	0.457±0.01	0.466±0.01	0.434±0.01	-
114	MQUm2-1	0.822±0.01	0.865±0.02	0.901±0.02	0.200±0.00
115	HZc1-3	0.448±0.01	0.439±0.00	0.231±0.00	-
116	JZc1-7	0.615±0.02	0.613±0.01	0.381±0.00	-
117	JZc1-8	0.503±0.01	0.500±0.01	0.442±0.00	-
118	QLm4-15	0.379±0.01	0.359±0.00	0.213±0.00	-
119	TZc1-10	0.473±0.10	0.472±0.04	0.523±0.05	-
120	TZc1-16	0.522±0.02	0.508±0.01	0.558±0.07	-
121	TZc1-6	0.469±0.01	0.484±0.01	0.054±0.00	-
122	TZc2-14	0.628±0.08	0.567±0.01	0.552±0.01	-
123	TZc2-8	0.346±0.00	0.345±0.01	0.279±0.01	-
124	TZc9-1	0.547±0.01	0.55±0.00	0.502±0.01	-
125	TZc9-11	0.547±0.00	0.545±0.01	0.562±0.01	-
126	TZc9-2	0.560±0.00	0.553±0.00	0.560±0.01	-
127	TZc9-3	0.454±0.01	0.454±0.00	0.093±0.00	-

Values are mean ± SD (n = 3). “-” indicates that the strain did not grow.

Table S3. Results of the determination of five antioxidant indexes in 26 lactic acid bacteria.

Strain	ABTS radical (%)			Hydroxyl radical (%)			Superoxide anion radical (%)			DPPH radical (%)			Reducing activity (OD)		
	CFS	IC	CFE	CFS	IC	CFE	CFS	IC	CFE	CFS	IC	CFE	CFS	IC	CFE
TZc2-21	84.21±0.10	32.12±5.15	14.67±2.10	84.15±0.61	21.29±3.89	10.68±0.76	26.57±0.68	22.3±0.90	25.27±0.49	96.08±0.27	36.21±0.56	23.14±1.09	0.78±0.05	ND	ND
TZc2-22	89.79±0.18	36.46±0.79	08.31±2.68	98.93±0.45	15.83±3.06	13.63±2.43	22.60±0.21	17.13±0.66	13.39±3.26	104.6±0.47	29.85±1.31	15.20±3.92	0.69±0.10	ND	ND
QL01	82.58±0.05	45.36±0.18	17.31±2.17	99.43±0.30	17.60±0.84	15.09±0.60	42.91±0.86	28.33±1.33	25.61±0.82	94.39±4.36	29.19±4.54	21.90±1.74	1.15±0.01	ND	ND
MQUy9-3	77.50±0.72	39.60±3.10	11.81±5.37	73.41±5.24	82.39±5.86	2.69±0.64	40.93±1.60	25.47±0.73	24.51±0.60	96.64±1.33	28.09±4.65	18.32±0.31	0.44±0.03	ND	ND
TZc1-2	90.13±0.04	78.51±0.81	49.07±0.94	99.56±0.23	34.12±1.79	19.25±0.04	20.98±1.77	21.67±1.54	22.49±1.94	107.87±2.50	38.33±4.42	26.46±0.61	0.22±0.02	ND	ND
HZc1-1	82.59±0.14	56.66±4.73	40.25±0.51	101.02±0.07	16.22±3.20	15.55±0.85	25.34±7.44	22.44±1.5	27.16±1.60	96.07±0.10	38.42±0.62	22.23±2.74	0.28±0.01	ND	ND
LQm2-4	84.48±0.05	65.57±7.93	42.70±3.04	101.65±0.03	40.34±4.56	18.49±2.49	24.97±1.10	20.74±0.15	22.64±1.21	95.71±0.28	37.95±2.97	28.40±1.41	0.53±0.06	ND	ND
XHc1-10	77.85±0.11	69.74±2.50	33.61±1.00	101.13±0.02	24.18±3.26	10.44±0.95	37.50±2.18	23.50±1.15	27.03±0.14	96.28±0.31	31.81±3.70	25.41±2.55	0.28±0.02	ND	ND
MQUc9-10	80.66±0.14	66.16±3.52	48.90±2.94	101.87±0.33	22.96±0.75	7.35±2.17	35.91±0.49	29.00±1.04	25.44±0.81	95.84±0.36	35.56±0.11	27.46±2.91	0.37±0.02	ND	ND
XHm5-8	80.69±0.10	54.88±0.51b	33.96±1.15	101.34±0.10	34.15±3.31	ND	35.57±0.41	25.31±1.17	21.88±0.79	96.96±0.25	37.31±3.22	29.00±0.77	0.32±0.02	ND	ND
TZc2-5	81.15±0.11d	67.21±8.38	23.82±0.87	101.34±0.17	30.65±0.50	3.89±1.33	31.90±1.02	18.24±1.59	14.24±0.44	91.97±0.37	35.78±3.45	31.11±0.59	0.36±0.03	ND	ND
ZNc1-6	80.53±0.21	58.01±5.00	33.36±1.79	102.45±0.23	30.33±1.73	2.94±1.82	41.00±0.86	28.77±1.58	24.11±0.68	94.90±0.29	36.06±2.76	24.19±4.42	0.33±0.03	ND	ND
TDc2-1	81.12±0.13	55.75±4.65	29.42±1.91	101.61±0.06	28.26±0.07	5.15±1.49	32.12±0.17	19.01±2.26	13.34±2.24	98.59±4.95	41.14±2.78	25.44±1.09	0.44±0.01	ND	ND
ZNc1-5	89.70±0.08	64.26±2.48	23.32±0.61	99.13±0.70	24.72±2.58	14.78±4.46	24.17±0.59	21.13±2.47	18.53±0.67	103.45±0.71	20.05±0.99	16.73±8.95	0.52±0.06	ND	ND
XHm4-4	82.03±0.11	28.79±1.71	11.71±0.29	62.42±0.36	24.38±1.11	14.77±0.50	39.72±1.05	23.33±1.28	23.73±1.70	97.70±0.84	34.94±2.82	26.43±4.49	0.33±0.06	ND	ND
XHm4-2	82.29±0.20	63.07±3.88	59.00±0.98	86.37±0.98	25.33±3.10	19.52±1.48	32.18±0.43	22.74±1.45	17.39±1.50	96.13±0.28	40.74±0.73	25.71±4.09	0.34±0.02	ND	ND
XHm1-6	83.89±0.12	62.71±3.35	69.44±1.07	94.82±0.94	16.84±1.11	9.85±2.46	26.11±1.15	20.83±1.62	22.23±0.89	96.44±0.47	42.57±4.74	28.08±2.36	0.57±0.09	ND	ND
HNy5-5	84.26±0.15	59.06±4.43	49.46±8.24	81.82±1.98	17.54±0.77	15.18±1.60	23.07±2.19	19.56±2.18	21.22±0.37	96.07±0.63	39.77±4.70	35.55±0.63	0.56±0.09	ND	ND
LQm2-7	77.99±0.16	62.10±0.39	57.03±1.34	101.29±0.11	12.40±2.00	12.42±0.30	37.83±2.95	27.16±0.36	22.22±0.65	96.40±0.06	34.49±1.25	30.78±3.45	0.21±0.00	ND	ND
GDm2-1	81.04±0.67	42.75±0.32	43.92±6.08	95.50±0.38	17.46±4.44	11.24±0.18	31.75±2.51	20.57±0.71	14.68±0.39	94.55±2.30	49.09±6.12	41.83±3.43	0.33±0.02	ND	ND
MQUm2-1	89.85±0.08	79.15±0.92	61.17±1.32	99.73±0.10	64.19±0.36	16.16±2.18	25.67±1.06	16.62±1.36	17.34±1.35	103.21±0.64	44.20±4.67	27.70±3.84	0.22±0.02	ND	ND
TDc2-4	82.36±0.11	76.73±1.41	73.19±0.79	101.73±0.08	16.71±2.16	16.42±0.27	32.24±2.70	24.10±0.52	22.39±2.51	96.11±0.16	31.17±1.20	24.66±4.09	2.32±0.03	0.21±0.01	ND
XHm4-1	84.49±0.09	66.98±2.51	58.37±2.81	101.31±0.13	25.92±1.11	17.56±2.14	23.01±0.86	20.02±0.18	19.64±1.70	95.75±0.75	36.79±3.19	27.67±0.72	1.15±0.21	0.20±0.04	ND

HZy3-1	78.25±0.18	73.67±0.36	67.45±0.97	100.98±0.09	51.51±2.21	7.42±2.33	36.03±2.07	27.62±0.98	21.81±0.64	95.79±0.42	31.92±1.55	22.8±1.16	0.25±0.01	0.21±0.00	ND
TZc1-3	80.98±0.22	64.79±3.71	53.63±0.22	100.98±0.17	24.96±6.76	8.01±3.78	36.00±2.66	21.84±2.14	18.47±1.00	95.99±0.67	44.94±0.53	32.98±1.24	0.25±0.01	0.14±0.00	ND
XHm3-3	81.88±0.22	61.32±2.52	48.35±4.31	101.16±0.03	27.92±1.20	43.00±2.47	34.32±1.34	24.12±1.04	18.16±1.08	98.31±3.77	34.74±1.77	24.06±5.69	0.29±0.05	0.15±0.00	ND

CFS: Cell-free fermentation supernatants; IC: Intact cells; CFE: Cell-free extracts. ND: Not detected. $ABTS^+$: Assay of scavenging of $ABTS^+$ free radical; OH^-: Assay of scavenging activity against hydroxyl radical, O^{2-}: Assay of scavenging activity against superoxide anion radicals; DPPH: Assay of scavenging of DPPH free radical; RA: Assay of reducing activity. Values are mean ± SD (n = 3).

Table S4. Putative virulence factors in the *Lactobacillus plantarum* QL01 genome.

Gene ID	Gene	Predicted functions	Identity (%)
GE000681	hasC	UDP-glucose pyrophosphorylase	75.34
GE000711	clpP	ATP-dependent Clp protease proteolytic subunit	69.79
GE001041	cpsI	UDP-galactopyranose mutase	65.03
GE001554	msrA/B(pilB)	trifunctional thioredoxin/methionine sulfoxide reductase A/B protein	64.03
GE001703	cpsA	undecaprenyl diphosphate synthase	63.22
GE001300	gnd	6-phosphogluconate dehydrogenase	62.87
GE001062	cpsI	UDP-galactopyranose mutase	62.24
GE001110	clpE	ATP-dependent protease	60.68
GE002978	lap	Listeria adhesion protein Lap	58.78
GE000657	htpB	Hsp60, 60K heat shock protein HtpB	58.17
GE000904	clpC	endopeptidase Clp ATP-binding chain C	58.13
GE001036	cps4I	UDP-N-acetylglucosamine-2-epimerase	56.46
GE001084	iap/cwhA	P60 extracellular protein, invasion associated protein Iap	54.55
GE000306	lap	Listeria adhesion protein Lap	53.03
GE002399	iap/cwhA	P60 extracellular protein, invasion associated protein Iap	52.94
GE002318	gtcA	wall teichoic acid glycosylation protein GtcA	52.42
GE000966	psaA	manganese ABC transporter, manganese-binding adhesion liprotein	51.31

Note: only listed the annotated genes with more than 50% similarity.
